# Cell-cycle dependence on the biological effects of boron neutron capture therapy and its modification by polyvinyl alcohol

**DOI:** 10.1038/s41598-024-67041-6

**Published:** 2024-07-19

**Authors:** Yusuke Matsuya, Tatsuhiko Sato, Tamon Kusumoto, Yoshie Yachi, Ryosuke Seino, Misako Miwa, Masayori Ishikawa, Shigeo Matsuyama, Hisanori Fukunaga

**Affiliations:** 1 Nuclear Science and Engineering Center, Japan Atomic Energy Agency, Tokai, 319-1195 Japan; 2 Faculty of Health Sciences, Hokkaido University, Sapporo, 060-0812 Japan; 3 National Institutes for Quantum and Radiological Science and Technology, Chiba, 263-8555 Japan; 4 Graduate School of Health Sciences, Hokkaido University, Sapporo, 060-0812 Japan; 5 Department of Quantum Science and Energy Engineering, Graduate School of Engineering, Tohoku University, Sendai, 980-8579 Japan; 6 Center for Environmental and Health Sciences, Hokkaido University, Sapporo, 060-0812 Japan; 7 Institute of Development, Aging and Cancer, Tohoku University, Sendai, 980-8575 Japan

**Keywords:** BNCT, BPA, Cell cycle, CR-39 detector, Heavy ions, PVA, Biophysics, Cell biology, Mathematics and computing

## Abstract

Boron neutron capture therapy (BNCT) is a unique radiotherapy of selectively eradicating tumor cells using boron compounds (e.g., 4-borono-l-phenylalanine [BPA]) that are heterogeneously taken up at the cellular level. Such heterogenicity potentially reduces the curative efficiency. However, the effects of temporospatial heterogenicity on cell killing remain unclear. With the technical combination of radiation track detector and biophysical simulations, this study revealed the cell cycle-dependent heterogenicity of BPA uptake and subsequent biological effects of BNCT on HeLa cells expressing fluorescent ubiquitination-based cell cycle indicators, as well as the modification effects of polyvinyl alcohol (PVA). The results showed that the BPA concentration in the S/G_2_/M phase was higher than that in the G_1_/S phase and that PVA enhances the biological effects both by improving the uptake and by canceling the heterogenicity. These findings might contribute to a maximization of therapeutic efficacy when BNCT is combined with PVA and/or cell cycle-specific anticancer agents.

## Introduction

The therapeutic potential of neutron capture was first proposed in 1936, just a few years after neutron’s discovery^1^. Among the modalities using photons, electrons, and light and heavy ions, boron neutron capture therapy (BNCT) has a unique feature in that it uses short-range heavy ions (such as α-particles and Li ions) emitted by the ^10^B(n,α)^7^Li reaction, which can selectively eradicate cells that take up tumor-seeking ^10^B compounds^2^. To date, experimental and clinical studies on BNCT have shown great potential in eradicating several cancers, such as melanoma, head and neck cancers, and brain tumors, while minimizing damage to normal tissues^3^.

Clinical trials on BNCT began in 1951 for the treatment of malignant glioma^4,5^; however, in the early days of the study, serious adverse effects such as brain necrosis were reported because of technical limitations. A breakthrough was the development of a tumor-seeking boron compound, 4-borono-l-phenylalanine (BPA), which can be highly taken up in tumors compared with the surrounding normal tissues^6^. Furthermore, the use of BPA clustered by polyvinyl alcohol (PVA-BPA) has shown the potential to enhance the therapeutic effects of BPA^7^. Along with effective ^10^B delivery techniques, another breakthrough was the development of an accelerator-based (AB) neutron source in clinical facilities, which can generate high-intensity epithermal neutrons (> 1 × 10^9^ n/cm^2^/s) at the beam aperture^8^. Therefore, AB-BNCT with BPA is expected to immediately spread to clinical facilities worldwide^9^.

For AB-BNCT, intravenous injection of BPA is essential^10^; however, its ability to deliver drugs to tumors remains a challenge because intracellular BPA concentration varies unevenly in terms of space and time^11^. Regarding the temporal dynamics of BPA, we previously investigated the time dependencies of ^10^B distribution in melanoma and found that the effects of cell recovery (DNA lesion repair) during exposure to neutrons are reduced compared with photon beams but still not negligible when delivering high doses to tumors^12^. In addition, the biological effects resulting from the spatial heterogeneity of BPA have yet to be fully elucidated. Previously, we reported that the spatial heterogeneities of ^10^B distribution are intrinsically associated with the curative effects following BNCT^13^. The cell cycle is a possible biological factor inducing such spatiotemporal heterogeneous ^10^B distribution^14^. Thus, in this study, we attempted to elucidate the cell cycle dependency of the BPA and PVA-BPA distribution and its possible biological effects on the therapeutic efficacy of AB-BNCT.

**Figure 1:**
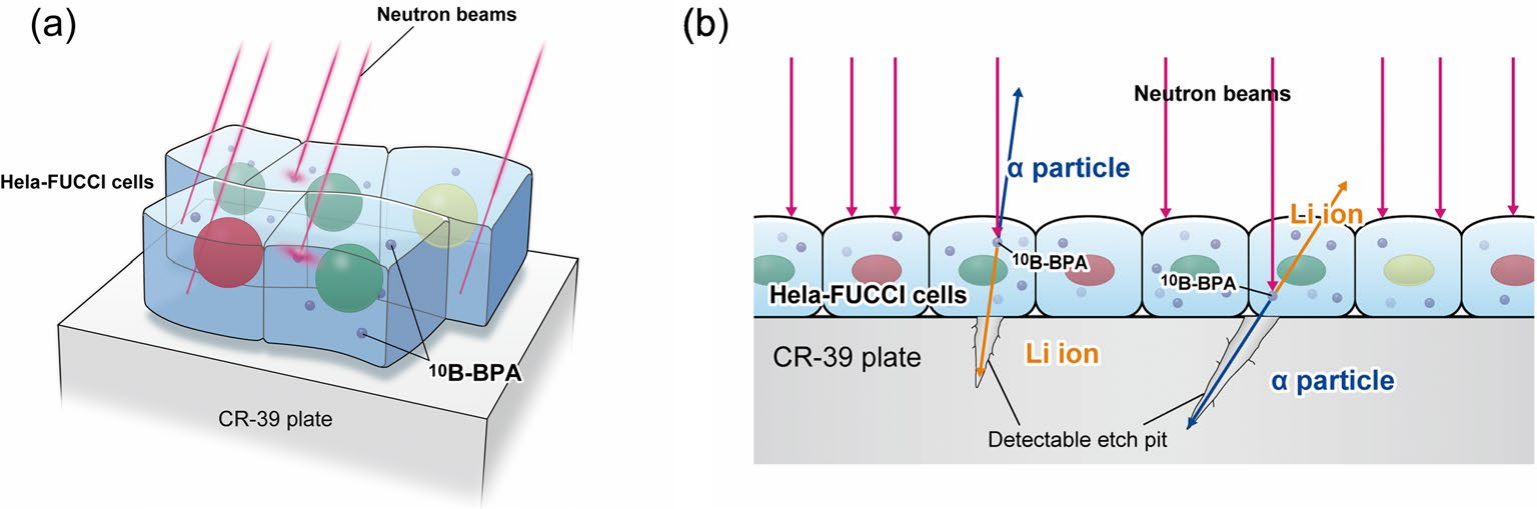
Schematic illustration of the experimental design: (**a**) depicts the HeLa-FUCCI cells plated on the CR-39 plastic detector. (**b**) details the detection of α-particles and Li-ions generated by ^10^B(n,α)^7^Li reactions. The red, yellow, and green cell nuclei represent the G_0_/G_1_ phase, early S phase, and S/G_2_/M phase, respectively.

To experimentally determine the heterogeneities of BPA concentration at the cellular level, the CR-39 plastic nuclear track detector was used^15,16^, which detects α-particles and Li ions generated by ^10^B(n,α)^7^Li reactions without background noise from photons and electrons (Fig. 1a)^17^. The damaged regions along the ion’s path are preferentially etched by chemical treatments using alkaline solutions (e.g., NaOH and KOH), and conical pores (so-called etch pits) are then formed (Fig. 1b). From the density of the etch pits, the intra and intercellular BPA distributions can be determined with a theoretical estimation of the number of ^10^B(n,α)^7^Li reactions^18^ using a Monte Carlo simulation for radiation transport. Cancer cells expressing the fluorescent ubiquitination-based cell cycle indicators (FUCCI)^19,20^, which enable the visualization of each phase of the cell cycle via fluorescent microscopy, were also used. Thus, as shown in Fig. 1, the cell cycle dependence of BPA uptake could be quantitatively evaluated. In addition to experimental data, the curative effects of BNCT for each phase of the cell cycle were identified using a biophysical model (so-called integrated microdosimetric–kinetic [IMK] model^21^), which allows the prediction of the surviving fraction in various cell cycle conditions and elucidation of biological factors to improve the curative effects. This interdisciplinary approach with radiation track detection, cell cycle analysis, and biophysical simulation enabled the generation of fundamental knowledge to improve AB-BNCT and establishment of effective drug delivery strategies.

## Materials and methods

### Cell culture

The study used two cell lines, namely, human cervical cancer (HeLa; RCB0007, RIKEN Science Institute BRC, Ibaraki, Japan) and HeLa expressing FUCCI (HeLa-FUCCI; RCB2812, RIKEN Science Institute BRC)^19,20^. HeLa-FUCCI cells were cultured in RPMI-1640 medium (Thermo Fisher Scientific Inc., Tokyo, Japan) with 10% fetal bovine serum (Nichirei Bioscience Inc., Tokyo, Japan) and 1% penicillin/streptomycin (p/s). The cells were maintained at 37 °C in a humidified atmosphere of 95% air/5% CO_2_.

### BPA and PVA-BPA preparation

Two boron compounds, namely, BPA and BPA with poly(vinyl alcohol) (PVA-BPA), were used. The BPA was provided as a powder by Stella Pharma Corporation (Osaka, Japan). The BPA powder was dissolved in sterile phosphate-buffered saline (PBS) (–), and a BPA solution was made. Meanwhile, PVA with an average degree of polymerization of 2000 and a degree of saponification of 98.9%/mol was purchased from Nakarai Tesque (Kyoto, Japan). The PVA powder and BPA powder were simultaneously dissolved into PBS (–) by heating, and PVA-BPA was prepared.

HeLa and HeLa-FUCCI cells were plated on a CR-39 plastic detector (BARYOTRAK, Nagase Landauer, Ltd.) and allowed to adhere overnight. After adherence, BPA was administered to the cells. First, the case of constant 600 ppm was selected, and the treatment period of BPA was changed, i.e., 30 min, 4 h, and 24 h, to check the time-dependent BPA uptake after injection. Given the dynamics of BPA uptake after injection, the BPA concentration increases approximately 2 h after injection^22^. Considering this, the BPA concentration of the culture medium was also changed from 6 to 6000 ppm for a treatment period of 4 h. Meanwhile, PVA-BPA was administered to cells at a final concentration of 0.02–0.2% PVA and 20 ppm BPA for 4 h. The effects of PVA on cell culture were also checked, from which a high density of 2% PVA is not suitable because PVA was too viscous. After removing the medium containing BPA and PVA-BPA, the cells were fixed in 4% paraformaldehyde for 10 min. In this study, the change in cell number and position on the CR-39 plate must be eliminated to detect the fluence of α-particles and Li ions at a micron scale. Considering this, the cells were washed with methanol to remove surface moisture and fixed.

### Irradiation with an AB neutron source

By using the Fast Neutron Laboratory at Tohoku University^23^, the accelerated 3.0-MeV protons with a beam current of 0.15 μA were incident on the Li metal target, and AB neutrons were generated. The dried cells on the CR-39 plate were irradiated with AB neutrons for 48 h. A schematic illustration of irradiating HeLa-FUCCI cells with the AB neutrons is shown in Fig. 1a, in which red, yellow, and green cell nuclei represent the G_0_/G_1_, early S, and G_2_/M phases, respectively. In the same manner as the HeLa-FUCCI, the HeLa cells were also irradiated with the neutrons. The α-particles and Li ions generated by ^10^B(n,α)^7^Li reactions were detected by the CR-39 detector. When irradiating the dried cells with AB neutrons, the 11 CR-39 plates were piled up and surrounded by the polyethene block (5 × 10 × 20 cm^3^) for efficient measurement of BPA concentration within cells. The experimental geometry is shown in Fig. S1 of Supplementary Material A.

**Figure 2:**
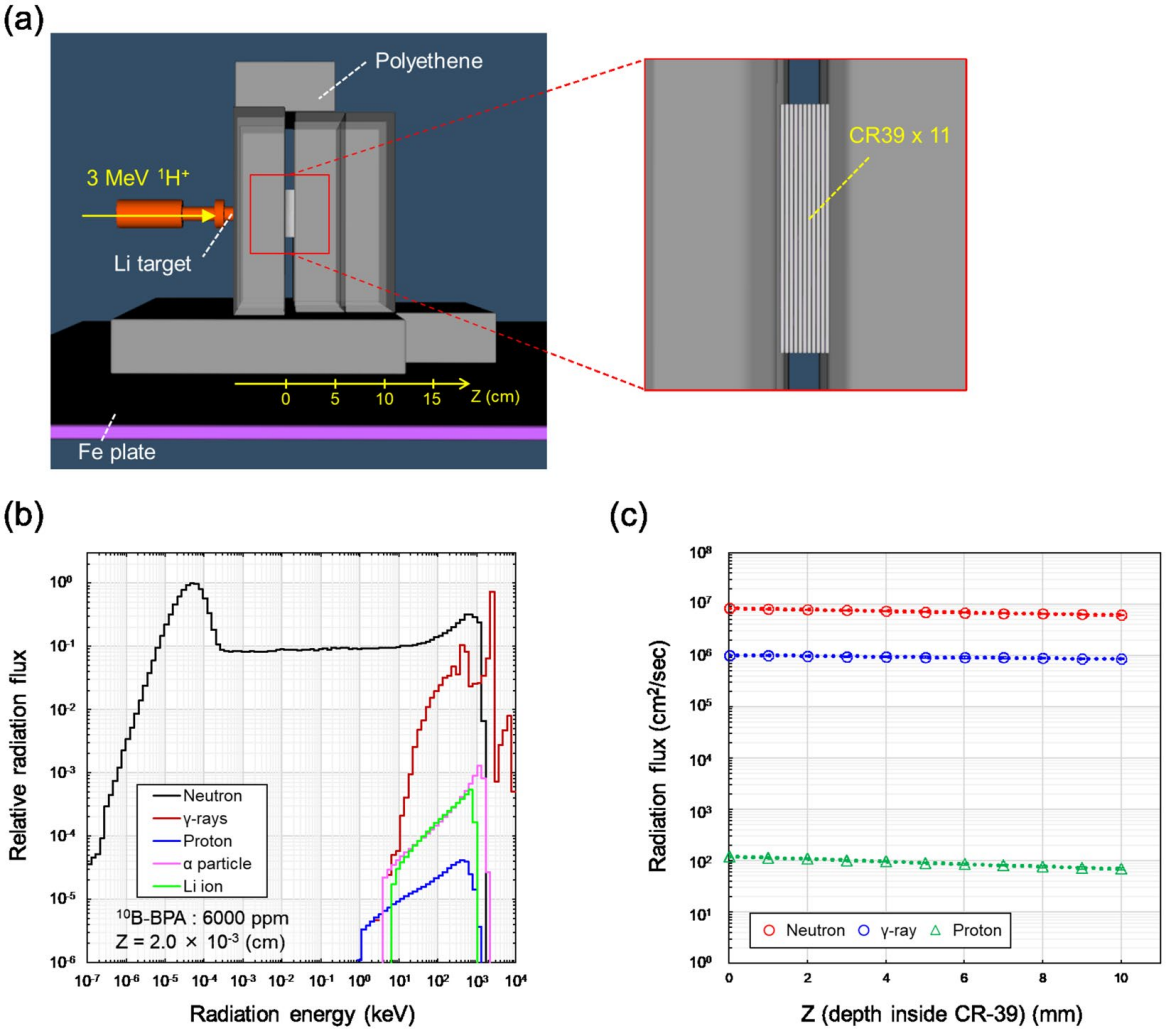
Irradiation geometry and the physical feature: (**a**) depicts the experimental geometry considered in the PHITS code; (**b**) refers to the radiation energy spectra at the position of the first CR-39 plate with 600 ppm BPA, which were calculated using the PHITS code^25^; and (**c**) shows the depth-dependence of radiation flux in this experiment. The three-dimensional (3D) geometry shown in (**a**) was illustrated by the PHIG-3D software^26^. In (**b**) and (**c**), the neutrons were generated by accelerating the protons into the Li metal target.

The flux of the thermal neutrons was measured using a scintillator and optical fiber (SOF)^24^. Meanwhile, a Monte Carlo simulation with the Particle and Heavy Ion Transport code System (PHITS) (version 3.29) was also performed^25^, and the total neutron flux incident on each CR-39 plate in this experiment was calculated using the [t-cross] tally, which enabled us to obtain the fluences in any specified surface in the PHITS code. Figure 2a shows the simulation geometry, which was designed to reproduce the experimental geometry shown in Fig. S1b of Supplementary Material A, which was depicted by the PHIG-3D software^26^. The simulation geometry considered in the PHITS code from a different view is illustrated in Fig. S2 of Supplementary Material A. In the simulation, the nuclear data library of the ^7^Li(p,n)^7^Be in the Japanese Evaluated Nuclear Data Library 4.0 high-energy file (JENDL-4.0/HE) was used^27,28^. The simulation accuracy of JENDL-4.0/HE implemented in the PHITS code has been verified in our previous report, in which the neutron energy spectra for each angle and the contents of thermal neutrons have been checked by comparing the recommended spectra calculated by the Fortran program LIYIELD.FOR and the experimental CD ratios measured using a boron trifluoride (BF_3_) detector^18^. In the simulations, the density of the polyethene (gray block in Fig. 2a) was assumed to be 0.935 g/cm^3^.

For radiation transport, the electron gamma shower mode^29^ and event generator mode were used^30^. The cutoff energies, except for the neutrons, were set to 1.0 keV, whereas those for neutrons were set to 1.0 × 10^−4^ eV. Figure 2b shows the energy spectra of neutrons, photons (γ-rays), recoiled protons, α-particles, and Li ions incident on the first CR-39 (z = 2.0 × 10^−3^ cm, Fig. 2a) containing cells administered with 600-ppm ^10^B. The radiation flux (trajectories) calculated using the PHITS code are shown in Fig. S2 of Supplementary Material A. The depth dependence of the radiation flux of neutrons, γ-ryas, and recoiled protons is depicted in Fig. 2c. The depth dependence of neutron and proton flux (Fig. 2c) should be noted when obtaining the boron densities within the cells.

### Chemical etching of CR-39 detector

CR-39 plastic nuclear track detectors enable the measurement of radiation tracks by detecting the damaged regions (so-called etch pits) preferentially etched relative to the undamaged regions by the chemical etching treatment using alkaline solutions (e.g., NaOH and KOH). Etch pits can be seen using an optical microscope above a certain LET threshold^15,16^. From such mechanisms of CR-39, recoiled protons, which are not desired etch pits in this study, are also detectable in BNCT irradiations. Considering these, to reduce the detection efficiency of recoiled protons, chemical etching treatments with potassium–hydroxide–ethanol–water solution with ethanol concentrations 15 wt.% (PEW-15) were adopted^31^.

For the PEW-15 solution, the detection efficiencies for α-particles and Li ions also decrease. The CR-39 detectors were irradiated with 6000 ppm BPA by the neutrons generated by 3.0 or 2.5 MeV protons for 1 h in Tohoku University^23^. Then, the densities of etch pits (/cm^2^) for 0 and 6000 ppm etched by the PEW-15 solution were measured and compared with those by 0wt.% (PEW-0) solution and the corresponding PHITS simulation. The etching time and temperature for the PEW-0 were 1613 min and 70 ℃, respectively. The microscopic images for the PEW-0 and PEW-15 are shown in Fig. S3 of Supplementary Material A. The detection efficiencies for nuclear reactions (α-particles and Li ions) and recoiled protons are shown in Fig. S3b and S3c, respectively. From Fig. S3b and S4c, the efficiencies of α-particles and Li ions for PEW-0 and PEW-15 were 92.9% ± 4.6% and 62.9% ± 2.8%, respectively, whereas those of recoiled protons for PEW-0 and PEW-15 were 57.8% ± 0.9% and 12.2% ± 0.2%, respectively.

After irradiating the CR-39 plates with the neutrons, the cell image was taken using the microscope (BZ-9000; Keyence, Osaka, Japan). To overlay the cell image on the etch pit after the etching treatment, some characteristic scars on the CR-39 were noted. After checking the scars, the cells were removed from the CR-39 with ethanol (FUJIFILM Wako Pure Chemical Corp., Osaka, Japan), and etching treatment with PEW-15 solution was performed at 50 ℃ for 280 min. Thereafter, etch pit images were taken using a microscope. The number of etch pits per cross-section of the cell was counted using Image J^32,33^.

### Calculation of BPA concentration from the etch pits

To obtain the BPA concentration within the cells, PHITS simulations were performed at the same experimental condition (i.e., the same BPA concentration as administered in the experiments), and the frequency of ^10^B(n,α)^7^Li reactions that occurred during the irradiation was counted, assuming that the BPA is fully taken up and is uniformly distributed within the cells (uptake fraction of 100%). Using the same irradiation geometry and the same physical features of neutrons as in Fig. 2, the fluences of -particles and Li ions at the surface between the cells and CR-39 plates were calculated using the [t-cross] tally. After the calculation, the ideal fluences and the experimentally obtained etch pits were compared, and the BPA concentration within cells was calculated. The number of etch pits correlated with the detection efficiency, i.e., 92.9% ± 4.6%.

### Biophysical model for predicting surviving fraction

By using the BPA concentration measured by the CR-39 detector, the cell killing, and RBE for the in vitro clonogenic cell survival were predicted based on the IMK model. In the previous IMK model development, overkill effects at high LET range were considered using the modified MK model^34^. In the IMK model, the cell nucleus (radiation target in cells) is subdivided into multiple micro-order territories (so-called domains) to incorporate microdosimetry^35^. The induction of potentially lethal lesions (PLLs), which correspond to DNA DSBs, transformation from PLLs to non-repairable lesions (so-called lethal lesions [LLs]), and repair dynamics were modeled using the kinetic equations. Based on the dynamics, the surviving fraction [S_*_$\text{(}{D}\text{)}$] as a function of absorbed dose *D* for a certain cell cycle phase can be expressed by the following equation:

1 $$\begin{aligned} - {\text{ln}} S_{{ \star }} (D)\, & { = }\left( {\alpha_{{{0}{ \star }}} + \frac{{y^{*} }}{{{{r\pi r}}_{{\text{d}}}^{{2}} }}\beta_{{ \star }} } \right)D{ + }\beta_{{ \star }} D^{{2}} \\ & = \left( {\alpha_{{{0}{ \star }}} + z_{{{\text{1D}}}}^{*} \beta_{{ \star }} } \right)D + \beta_{{ \star }} D^{{2}} \\ & { = }\alpha_{{ \star }} D + \beta_{{ \star }} D^{{2}} \\ \end{aligned}$$

where $$\alpha_{{{0}{ \star }}}$$ and $$\beta_{{ \star }}$$ represent the coefficients to dose *D* and dose square *D*^2^. In this study, these parameters were defined as cell cycle-specific values. $${ \star }$$ represents certain cell cycle phase (i.e., G_1_/S or S/G_2_/M), $$z_{{{\text{1D}}}}^{*} \, = \,y^{*} {/{\rho\pi r}}_{{\text{d}}}^{{2}} ,{ }\alpha_{{ \star }}$$ = $${ }\alpha_{{{0}{ \star }}} + \,z_{{{\text{1D}}}}^{*}$$$$\beta_{{ \star }}$$, where $$\uprho$$ and *r*_d_ are the density and radius of the domain, respectively, i.e., $$\rho$$ = 1.0 g/cm^3^ and *r*_d_ = 0.5 μm. Moreover, microdosimetric quantity $$y^{*}$$ can be given by

2 $$y^{*} { =\,\, }\frac{{y_{{0}}^{{2}} \int {\left[ {{1 } - {\text{exp}}\left( {{ - }y^{{2}} {/}y_{{0}}^{{2}} } \right)} \right]} f\left( y \right){\text{d}}y}}{{\int {yf\left( y \right){\text{d}}y} }}$$

where *y* is the lineal energy (keV/μm), *f*(*y*) is the probability density of *y*, and *y*_0_ is a so-called saturation parameter to express the overkill effect, which was set to 250 keV/μm in this study. ${y}^{*}$ can be calculated using the Monte Carlo simulation code of PHITS, whereas the parameter set (${\alpha}_{{0}\star }$, ${\beta}_{\star }$) can be determined by fitting Eq. (1) to the experimental dose–response curve of the surviving fraction.

Basically, Eq. (1) was fitted to the experimental dose–response curves of cell survival for G_1_/S and S/G_2_/M phases, and the biological effect by BNCT irradiation were discussed. In general, the biological effects for the asynchronous phase are evaluated to determine the curative effects. Thus, based on the previous modeling for heterogenous cell populations, the surviving fraction for the asynchronous phase can be expressed by

3 $$S(D{) = }f_{{{\text{G}}_{{1}} {\text{/S}}}} { }S_{{{\text{G}}_{{1}} {\text{/S}}}} {(}D{)} + f_{{{\text{S/G}}_{{2}} {\text{/M}}}} S_{{{\text{S/G}}_{{2}} {\text{/M}}}} {(}D{)}$$

where ${ }f_{{{\text{G}}_{{1}} {\text{/S}}}}$ and $f_{{{\text{S/G}}_{{2}} {\text{/M}}}}$ are the fractions of G_1_/S and S/G_2_/M phases, and $S_{{{\text{G}}_{{1}} {\text{/S}}}} {(D)}$ and $S_{{{\text{S/G}}_{{2}} {\text{/M}}}} {(D)}$ are the surviving fractions of G_1_/S and S/G_2_/M phases, respectively. Here, $f_{{{\text{G}}_{{1}} {\text{/S}}}}$ and $f_{{{\text{S/G}}_{{2}} {\text{/M}}}}$ can be obtained from the cell experiments (e.g., FUCCI system or flow cytometric analysis with propidium iodide [PI]). By using Eqs. (1) and (3), the present model can provide the RBE for the in vitro clonogenic cell survival.

### Mean inactivation doses and the RBE

As shown in Eq. (3), the surviving fraction can be expressed using the complex formula as the absorbed dose. To evaluate the effect by BPA uptake on cell survival, the concept of the mean inactivation dose $\overline{D}$^36^ recommended by ICRU Report 30 was used^37^. In this concept, dose–response curve, taking into account of the survival fraction *S*(*D*) as an integral probability distribution, the mean dose necessary to inactivate cells (so-called mean inactivation dose) $\overline{D}$ is expressed by

4 $$\overline{D}{ = }\mathop \smallint \limits_{0}^{\infty } S(D{\text{)d}}D{.}$$

By using Eq. (4), the RBE for the mean inactivation dose can be given by

5 $${\text{ RBE = }}\frac{{\overline{D}_{{{\text{ref}}}} }}{{\overline{D}}}$$

where $\overline{D}$_ref_ and $\overline{D}$ are the mean inactivation doses of the reference radiation (i.e., ^60^Co γ-rays) and any radiation, respectively.

### Estimation of the RBE based on the MK model and PHITS

By using the MK model, the cell surviving fraction after BNCT irradiation was estimated, and the effect of the cell cycle on the biological effects of HeLa (HeLa-FUCCI) cells was discussed. First, using the [t-sed] tally of PHITS, which can calculate the distribution of energy deposited in the domain in the macroscopic Monte Carlo simulation^38,39^, the lineal energy distributions for 150 kVp X-rays were calculated. By using the calculated *y* distributions and Eq. (2), the y^*^ necessary for predicting SF and RBE in the MK model was calculated. Then, the parameter sets (${\alpha}_{{0}\star }$, ${\beta}_{\star }$) in the MK model for the G_1_/S and S/G_2_/M phase HeLa cells were determined by fitting Eq. (1) to the experimental survival data^40^ using MCMC simulation^41^. The MCMC simulation allows the estimation of the uncertainties of model parameters based on Bayesian estimation, and the details of this MCMC algorithm are provided previously^41^. When performing the MCMC simulation, a uniform distribution for $\alpha_{{{0}{ \star }}}$ and $\beta_{{ \star }}$ as the prior distribution was assumed. The set of model parameters $\theta$($\alpha_{{{0}{ \star }}}$, $\beta_{{ \star }}$) was sampled following the likelihood *P*(*d*_*i*_|*θ*) and transition probability *α*_P_ as follows:

6 $${ }P(d|\theta ) = \mathop \prod \limits_{i = 1}^{N} [P(d_{i} |\theta ){] = }\mathop \prod \limits_{i = 1}^{N} \left\{ {\frac{{1}}{{\sqrt {{{2\pi \sigma }}} }}{\text{ exp}}\left[ { - \frac{{{(} - {\text{ln }}S_{{\text{expi }}} {\text{ + ln }}S_{{{\text{cali}}}} {)}^{{2}} }}{{{2}\sigma^{{2}} }}} \right]} \right\}$$

7 $$\alpha _{P} ~ = ~\frac{{P\left( {\theta ^{{{\text{candidate}}}} |~d} \right)}}{{P(\theta ^{{(t)}} |d)}}$$

where *d*_*i*_ (*i* = 1〜*N*) is the experimental surviving fraction [*d*_*i*_ = (*D*_*i*_, − ln *S*_exp*i*_)], *S*_exp_ is the experimental surviving fraction, *S*_cal_ is the surviving fraction calculated by the MK model, and *P*(*θ*|*d*) and* P*(*θ*^*candidate*^|*d*) are the posterior likelihood for the candidate (*t* + 1)-th and previous (*t*)-th conditions. After obtaining the $\theta$($\alpha_{{{0}{ \star }}}$, $\beta_{{ \star }}$), to estimate the SF and RBE of BNCT, the clinical neutron spectra in the BNCT facility based on a 2.5-MeV proton accelerator on Li target reported in the literature were used^42^. The neutrons were incident on the cell culture dish containing a medium with cells, and the y^*^ values were calculated by the [t-sed] tally. After that, by using the y^*^ value, model parameters, and Eqs. (1–5), the dose–response curve of cell survival for various radiation qualities and the RBE values of BNCT with BPA and PVA-BPA were estimated based on the BPA concentration measured by the CR-39 detector in this study. In addition, the surviving fraction was estimated assuming that the BPA was fully taken up in cells (i.e., administered BPA concentration = BPA concentration in the cells). After comparing them, we evaluated the effect of heterogeneous BPA uptake on biological effects.

### Clonogenic survival assay

To validate the model for predicting the surviving fraction for the asynchronous phase (i.e., Eqs. [1–3]), the cell surviving fraction of asynchronous HeLa-FUCCI cells was measured by a clonogenic assay as previously described^43^. Cells were plated and allowed to adhere overnight before irradiation. The HeLa-FUCCI cells were irradiated at room temperature with 150-kVp X-rays (1-mm Al filtration and high dose rate ≥ 1.82 Gy/min) using an X-ray generator (MBR-1520R-4, Hitachi Medical Co., Tokyo, Japan). After irradiation, the cells were incubated for 10–14 days. Colonies were fixed with methanol and stained with 2% Giemsa solution (Kanto Chemical Co. Inc.). Then, the surviving fraction, that is, the ratio of the plating efficiency of the irradiated group to that of the nonirradiated group, was calculated.

### Statistics

The significant differences among mean values in boron concentration and boron uptake were evaluated by the t-test. In this study, the error bars of the experimental data were expressed by the standard error of mean. Meanwhile, the agreement between experimental survival and the corresponding estimation based on the MK model was evaluated by the *R*^2^ value.

## Results

### Uptake of BPA and its cell cycle dependence

To measure the BPA densities taken up in cells, HeLa-FUCCI cells cultured on the CR-39 plastic detector were fixed after BPA or PVA-BPA administration. The schematic illustration is shown in Fig. 1. The cells were irradiated with AB neutrons at the Fast Neutron Laboratory, Tohoku University^23^. Figure 2a depicts the irradiation geometry illustrated using the PHIG-3D software^26^, where the energy spectra of radiation particles on all CR-39 were evaluated using a general-purpose Monte Carlo code of the PHITS^25^ (Fig. 2b and c). Images of the actual experimental geometry and radiation flux calculated by PHITS are shown in Fig. S1 and S2 of Supplementary Material A, respectively. As presented in Fig. 2b, the radiation fields are mainly composed of thermal neutrons, which are suitable for ^10^B(n,α)^7^Li reactions. After the long-term irradiation, as previously reported^31^, chemical etching treatment with potassium–hydroxide–ethanol–water solution with ethanol concentrations of 15wt.% (PEW-15) was performed to detect the tracks of α-particles and Li ions.

**Figure 3:**
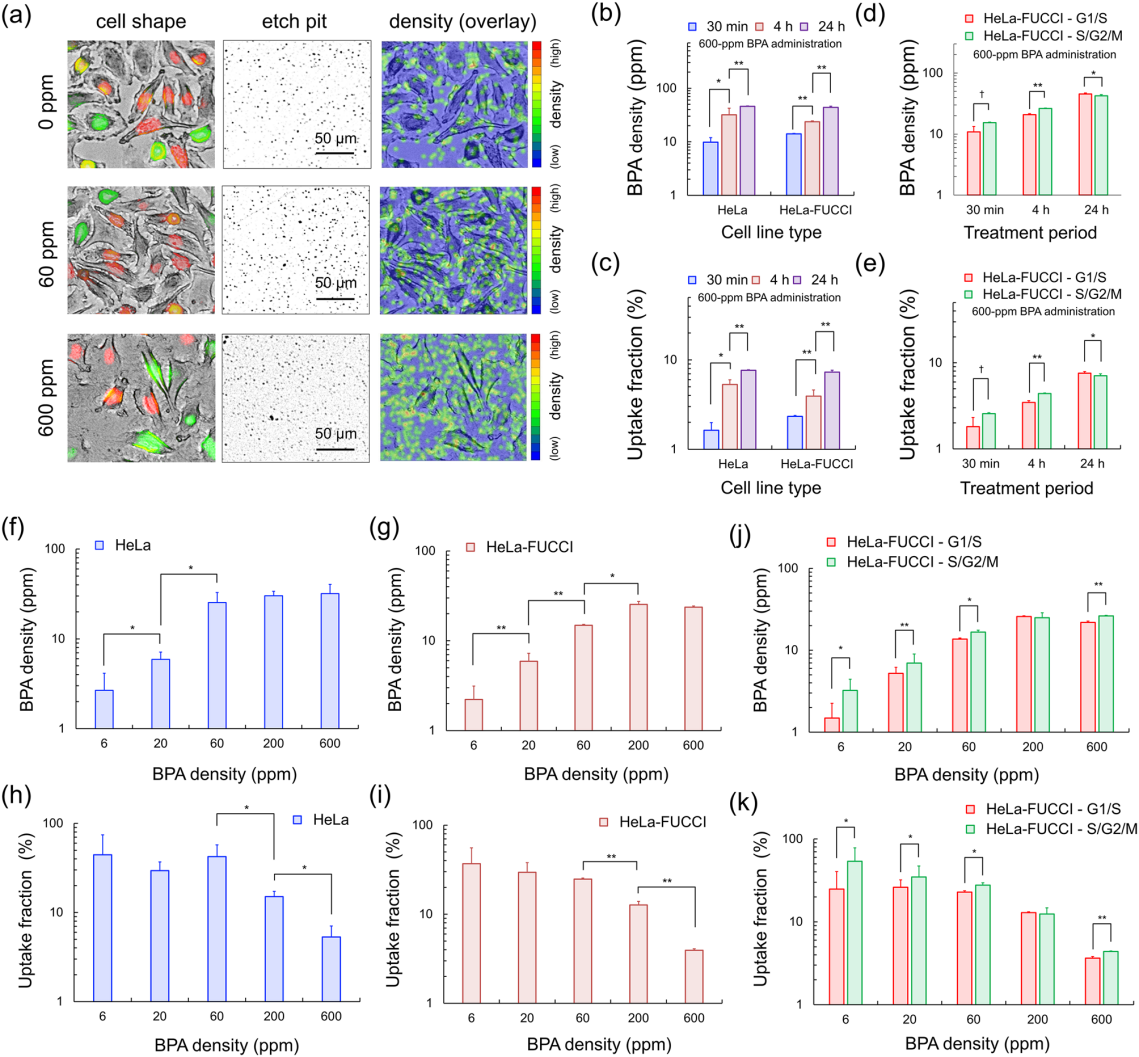
Boron concentration and uptake for BPA: (**a**) indicates the microscopic image of HeLa-FUCCI cells, etch pit image, and the overlay image for 0, 60, and 600 ppm. (**b**) and (**c**) represent the BPA concentrations and uptake fraction of HeLa and HeLa-FUCCI cells treated with 600-ppm BPA for 30 min, for 4 h, and for 24 h, respectively. (**d**) and (**e**) are the BPA concentrations and uptake fractions of G_1_/S and S/G_2_/M HeLa cells treated with 600-ppm BPA for 30 min, for 4 h, and for 24 h, respectively. (**f**)–(**i**) are the measured BPA concentrations and the uptake fraction against the administered concentration for HeLa and HeLa-FUCCI cell lines. (**j**) and (**k**) represent the the BPA concentration and uptake in G_1_/S and S/G_2_/M cells for various prescribed BPA concentrations. The error bar represents the standard error of the mean (s.e.m.). The sample number was at least two, and the number of analyzed cells was at least 100 for each administered BPA density. The symbols †,*, and ** indicate the 10%, 5%, and 1% significant difference, respectively.

Figure 3a depicts a microscopic image of HeLa-FUCCI cells, etch pit image, and overlay image at 0, 60, and 600 ppm. The etch pits in the G_1_/S (red and yellow in Fig. 3a) and /G_2_/M (green in Fig. 3a) phases were analyzed. The fractions of the cell cycle phases of HeLa-FUCCI and HeLa cells are shown in Fig. S4 of Supplementary Material A, in which BPA administration was found to be irrelevant to the cell cycle distribution. The density of the etch pits increases as the administered BPA density increases. The etch pits shown at 0 ppm represent the recoiled protons because CR-39 can detect protons. In this study, a PEW-15 solution was used; however, as the detection efficiency of protons was 12.2% ± 0.2% (Fig. S3), the recoiled protons could not be fully removed from the detection. Therefore, the number of etch pits per μm^2^ (etch pit density) was counted without the cells; then, the density of etch pits outside the cells was subtracted from those inside the cells. Moreover, the BPA concentration in the cells was obtained. The background of the etch pits by the recoiled protons and the sum of those by the recoiled protons and the ^10^B(n,α)^7^Li reactions are shown in Fig. S5 of Supplementary Material A.

By using this detection system, we first evaluated the time course of BPA uptake after the administration in increments of the number of etch pits per μm^2^ as shown in Fig. S5 of Supplementary Material A. Figure 3b and c shows the BPA concentrations and uptake fraction of HeLa and HeLa-FUCCI cells treated with 600-ppm BPA for 30 min, 4 h, and 24 h, respectively. The BPA concentration of both cell lines significantly increased in vitro as the treatment period increased. As shown in Fig. 3d and e, within 4 h after the treatment with BPA, the BPA concentration and uptake in the S/G_2_/M phase are significantly higher than those in the G_1_/S phase. Specifically, the significance was the highest 4 h after the administration. While the uptake fractions (the BPA density) of HeLa and HeLa-FUCCI at 24 h after the administration are the highest, the uptake of cells in the G_1_/S phase 24 h after administration was higher than that in the S/G_2_/M phase. This might be because the cells in the high-uptake S/G_2_/M phase progresses to the G_1_/S phase, judged from the doubling time (i.e., 45.7 ± 3.2 h^44^). In vivo, the BPA concentration peaked 2–3 h after intravenous injection^22^. We selected 4 h after administration when evaluating the concentrations of BPA and PVA-BPA for the subsequent experiments.

As shown in Fig. 3b and c, the BPA concentration taken up in cells was obtained for various prescribed BPA densities. The increase in the number of etch pits per cell following an increase in the prescribed BPA concentration is shown in Fig. S6 of Supplementary Material A. The measured BPA concentrations against the administered concentration are shown in Fig. 3f and g, in which HeLa and HeLa-FUCCI cells showed similar, and the BPA concentration of cells was saturated at ≥ 200 ppm. Figure 3h and i present BPA uptake as a function of the administered BPA concentration. The BPA uptake for a BPA administration concentration < 60 ppm is about 34.59% at constant; however, the uptake fraction monotonically decreases as the administered concentration is high. In the same manner as in Fig. 3d and e, as shown in Fig. 3j and k, the BPA concentration and uptake in the S/G_2_/M phase are significantly higher than those in the G_1_/S phase for administrations of various BPA concentrations. The histograms of the number of etch pits per μm^2^ are shown in Fig. S7 of Supplementary Material A, where an increase in the S/G_2_/M phase can be observed compared with those in the G_1_/S phase.

### Modification of cell cycle dependence of boron uptake by PVA-BPA

The cell cycle dependence of PVA-BPA on boron concentration in cells and RBE value was also investigated. Four concentrations of PVA, i.e., 0%, 0.02%, 0.2%, and 2%, were used, and they were prescribed to HeLa and HeLa-FUCCI cell lines. In the preliminary tests, the dried cells treated with 0%, 0.02%, 0.2%, and 2% PVA were observed, as shown in Fig. S8 of Supplementary Material A. From the observation, 2% PVA remained attached to the cells after fixation with PFA, which was not suitable for evaluating the uptake fraction of PVA-BPA. The pH of each PVA concentration was also measured. No dramatic differences were found between them (Fig. S9 of Supplementary Material A). As shown in Fig. S10 of Supplementary Material A, cell cycle analysis also revealed dramatic differences among 0%, 0.02%, 0.2%, and 2% PVA. Considering these results, the PVA at a final concentration of 0.02% and 0.2% was used for the cell experiment.

**Figure 4:**
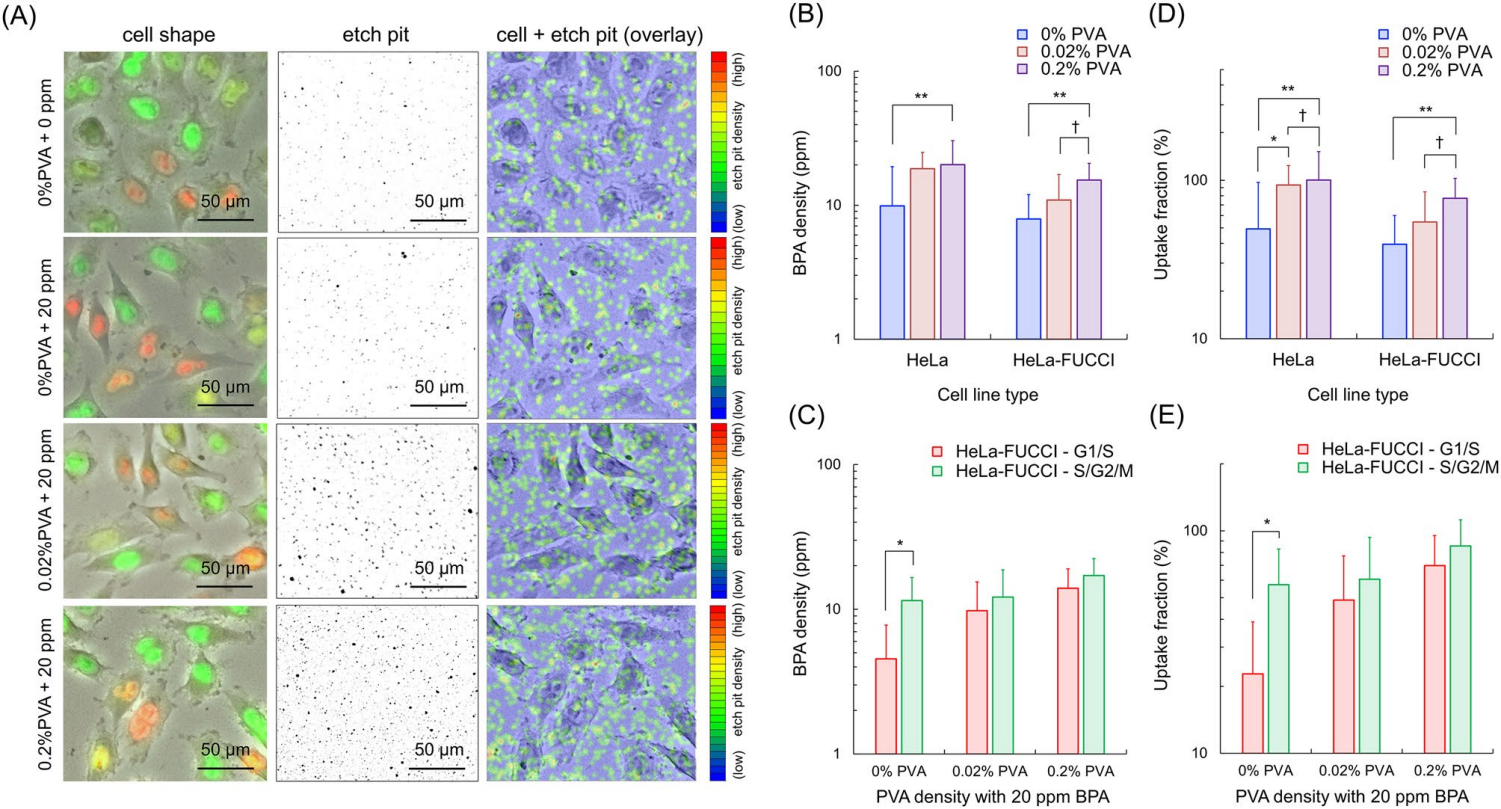
Boron concentration and uptake for PVA-BPA: (**a**) depicts the microscopic image of HeLa-FUCCI cells and the overlay image for various PVA-BPA concentrations. (**b**) and (**c**) represent the boron concentrations of HeLa and HeLa-FUCCI cells treated for 4 h. (**d**) and (**e**) indicate the uptake fractions of HeLa and HeLa cells treated for 4 h. The error bar represents the standard error of the mean (s.e.m.). The sample number was at least two, and the number of analyzed cells was at least 100 for each administered BPA density. The symbols †, *, and ** indicate the 10%, 5%, and 1% significant difference, respectively.

Figure 4a shows optical microscopic images of HeLa-FUCCI cells and the overlay image for 0% PVA with 0 ppm, 0% PVA with 20 ppm, 0.02% PVA with 20 ppm, and 0.2% PVA with 20 ppm. At a glance, the density of the etch pits of 0.2% PVA with 20 ppm is higher than that of 0% PVA with 20 ppm. Figure 4b and c show the BPA concentrations and uptake fraction of HeLa and HeLa-FUCCI cells treated with various PVA-BPA concentrations. Note that the treatment period of BPA or PVA-BPA was 4 h (Fig. 4). From Fig. 4b, the boron concentration within cells by PVA-BPA was at most about 2.0 times higher than that by only BPA, which indicates that the boron dose can be enhanced by PVA-BPA. In Fig. 4c, no significant differences were found in terms of boron concentration by PVA-BPA between the G_1_/S and S/G_2_/M phases. This result indicates that PVA can diminish the heterogeneous boron uptake induced by the cell cycle phase. Similarly, judging from no significant difference between the G_1_/S and S/G_2_/M phases in Fig. 4d and e, the uptake fraction can be improved by PVA-BPA, and the cell cycle dependence can be canceled.

### Estimation of curative effects by BNCT with BPA and PVA-BPA

**Figure 5:**
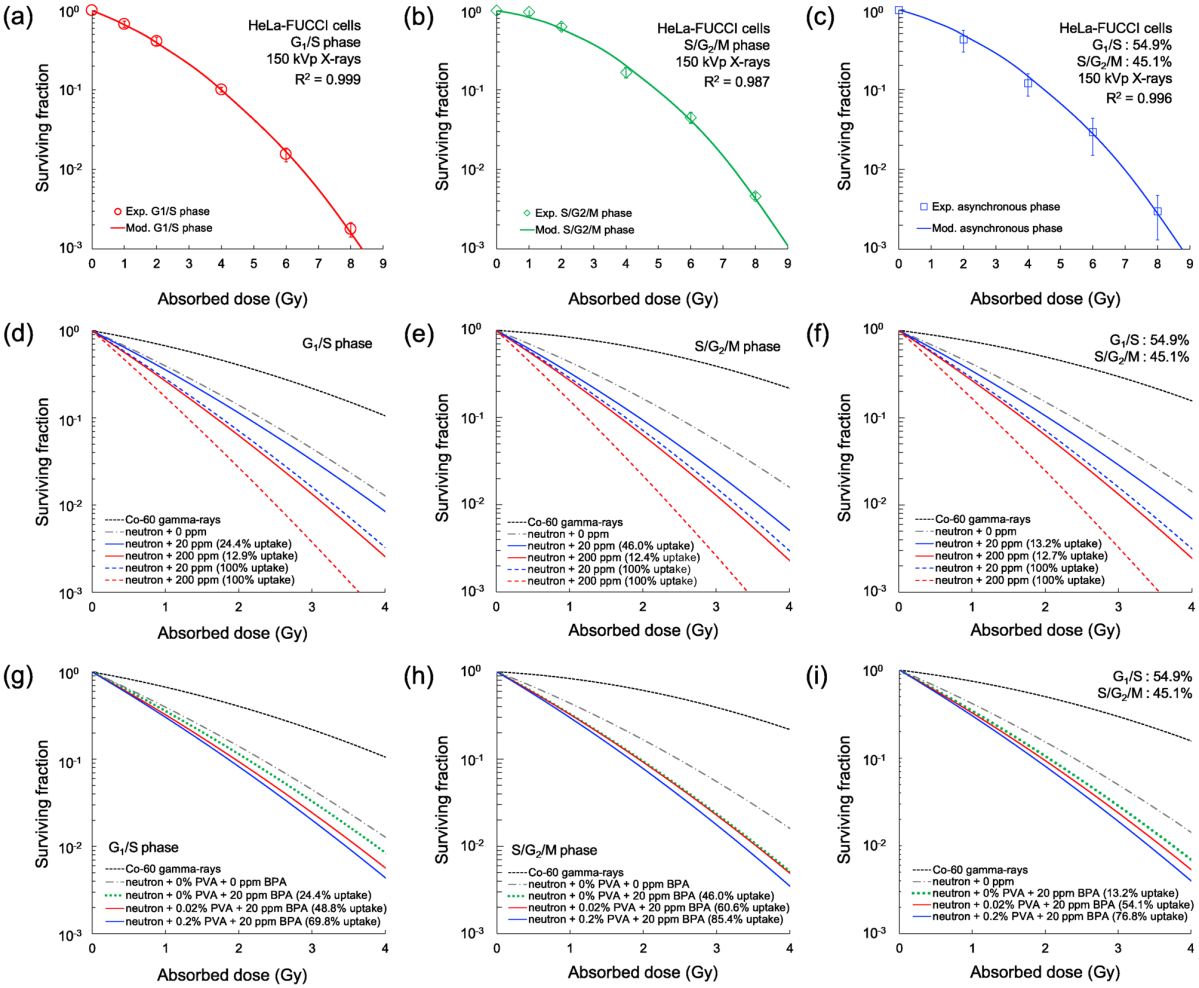
Dose–response curve of cell survival estimated by the IMK model: (**a**), (**b**) represent the fitting of the IMK model to the experimental survival of G_1_/S and S/G_2_/M phases, respectively. (**c**) depicts the comparison between the prediction using the present model (Eq. (3)) and the experimental data measured at asynchronous phase. Note that the experimental data was measured by means of clonogenic survival assay in this study. (**d**–**f**) depict the surviving fractions predicted based on the experimental BPA densities and those assuming the same densities as the administered those (uptake fraction = 100%) for the G_1_/S, S/G_2_/M, and asynchronous phases, respectively. (**g**–**i**) show the predicted surviving fractions for various PVA concentrations with 20 ppm BPA for the G_1_/S, S/G_2_/M, and asynchronous phases, respectively.

The cell cycle dependence of BPA and PVA-BPA densities were experimentally evaluated (Figs. 3 and 4). Next, we evaluated whether the effects of the ^10^B(n, α)^7^Li reactions were significant or not. To identify the curative effects, the IMK model (Eq. (1) in the Materials and Methods) was first fitted to the cell survival curve after X-ray irradiation to obtain the cell cycle-specific model parameters (${\alpha}_{{0}\star }$, ${\beta}_{\star }$) using the Markov chain Monte Carlo simulation (MCMC)^41^. Here, $\star$ represents a certain cell cycle phase, i.e., G_1_/S or S/G_2_/M). Figure 5a and b depicts the dose–response curve of cell survival in HeLa-FUCCI cells in the G_1_/S and S/G_2_/M phases, respectively, where symbols and lines are the corresponding experimental data^40^ and model prediction. The model parameters of HeLa-FUCCI cells in G_1_/S and S/G_2_/M phases are listed in Table 1. In the MCMC simulation, the value of the model parameters can be obtained as a distribution. The parameters set (${\alpha}_{{0}\star }$, ${\beta}_{\star }$) are shown in Fig. S11 of Supplementary Material B. To verify the IMK model used in this study, the dose–response curve of asynchronous HeLa-FUCCI cells was also predicted and compared with the corresponding measured data (Fig. 5c). From the comparison, the good agreement between them was shown, indicating that the concept of the IMK model is reasonable.

**Table 1:** Model parameters for predicting surviving fractions in the HeLa-FUCCI cell line.

Cell cycle	Parameter	mean ± sd	Unit	Note (meaning and how each is determined)
G_1_/S phase	*α*_0G1/S_	0.319 ± 0.093	Gy^−1^	Cell cycle-specific parameter determined from the experimental surviving fraction
	*β*_G1/S_	0.055 ± 0.012	Gy^−2^	Cell cycle-specific parameter determined from the experimental surviving fraction
	*z*_1D_*	0.698	Gy	Microdosimetric quantities of 150 kVp X-rays calculated by PHITS (*r*_d_ = 0.5 μm)
S/G_2_/M phase	*α*_0S/G2/M_	0.083 ± 0.177	Gy^−1^	Cell cycle specific parameter determined from the experimental surviving fraction
	*β*_S/G2/M_	0.067 ± 0.024	Gy^−2^	Cell cycle-specific parameter determined from the experimental surviving fraction
	*z*_1D_*	0.698	Gy	Microdosimetric quantities of 150 kVp X-rays calculated by PHITS (*r*_d_ = 0.5 μm)

By using the model parameters and microdosimetric quantities (i.e., lineal energy *y* [keV/μm]) calculated by the PHITS code^38,39^, the dose–response curve of the surviving fraction and RBE were estimated. Figure 5d–f show the dose–response curves of the survival of HeLa-FUCCI cells for various situations of BPA densities. In this simulation, ^60^Co γ-rays were selected as reference radiation (black-dotted line in Fig. 5d–f), whereas neutron irradiation was also depicted as the dose–response without boron effect (dash-dotted line in Fig. 5d–f). The neutron field at Tohoku University, which was used in this experiment, is not a BNCT facility. Considering this, in the subsequent calculation, the clinical AB-neutron spectra in the BNCT facility based on a 2.5-MeV proton accelerator on the Li target reported in the literature were used^42^, as shown in Fig. S12 of Supplementary Material B. When estimating the surviving fraction after BNCT irradiation for various BPA densities, the *y* distribution in the presence of ^10^B densities was calculated. The lineal energy distributions for ^60^Co γ-rays, AB neutrons, and BNCT (i.e., 20 and 200-ppm BPA) are depicted in Fig. S13 of Supplementary Material B. As shown in Fig. 5d–f, if the BPA densities within the HeLa cells are the same as the administered dose (hereafter called uptake fraction = 100%), the curative effects of BNCT are represented by the colored dotted lines. However, because of the uptake limit of the HeLa cells (the fractions are shown in Table 2), the cell killing effects predicted using the BPA densities measured in this study (colored solid lines) were lower than those in the case of uptake fraction (100%; colored dotted lines). The RBE values predicted based on the measured BPA densities in HeLa cells are summarized in Table 2, which shows that the RBE values were saturated at ≥ 200 ppm (2.51 ± 0.34 for 200 ppm in the G_1_/S phase and 3.46 ± 1.16 in the S/G_2_/M phase). The RBE as a function of the administered BPA concentrations is shown in Fig. S14 of Supplementary Material B, where the saturation of RBE values at high concentrations can be confirmed.

**Table 2:** RBE values predicted by the model based on the measured BPA uptake fractions.

Cell cycle phase	Boron compound	Administered BPA density	Uptake fraction (%)	RBE value
G_1_/S	BPA	0 ppm	–	1.85 ± 0.20
		20 ppm	24.4 ± 12.2	2.03 ± 0.23
		200 ppm	12.9 ± 0.3	2.51 ± 0.34
	0.02% PVA + BPA	20 ppm	48.8 ± 28.4	2.19 ± 0.27
	0.2% PVA + BPA	20 ppm	69.8 ± 25.4	2.30 ± 0.29
S/G_2_/M	BPA	0 ppm	–	2.37 ± 0.59
		20 ppm	46.0 ± 20.2	2.91 ± 0.84
		200 ppm	12.4 ± 2.3	3.46 ± 1.16
	0.02% PVA + BPA	20 ppm	60.6 ± 32.9	3.04 ± 0.91
	0.2% PVA + BPA	20 ppm	85.4 ± 26.7	3.23 ± 1.02
Asynchronous G_1_/S: 54.9% S/G_2_/M: 45.1%	BPA	0 ppm	–	2.10 ± 0.29
		20 ppm	13.2 ± 11.3	2.42 ± 0.37
		200 ppm	12.7 ± 1.1	2.94 ± 0.52
	0.02% PVA + BPA	20 ppm	54.1 ± 21.5	2.57 ± 0.41
	0.2% PVA + BPA	20 ppm	76.8 ± 18.4	2.71 ± 0.45

As for PVA-BPA, the dose–response curves of cell survival for 0.02% and 0.2% PVA with 20-ppm BPA are shown in Fig. 5g–i, in which the solid lines and the green-dotted line represent the curves of PVA-BPA and BPA administration, respectively. As shown in Figs. 3 and 4, increments in the uptake fraction of ^10^B concentrations by the use of PVA for the G_1_/S, S/G_2_/M, and asynchronous phases (Table 2) were observed. The IMK model prediction suggests that cell killing can be enhanced by PVA. The increments of the mean RBE value by the use of PVA in the G_1_/S and S/G_2_/M phases were 0.16–0.27, 0.13–0.32, and 0.12–0.24, respectively. Focusing on the uptake fractions of BPA in Table 2, the RBE values of the G_1_/S phase were lower than those of the S/G_2_/M phase, suggesting the insufficient enhancement of radiosensitivities under irradiation with high linear energy transfer (LET) ions (i.e., α-particles and Li ions) in the G_1_/S phase. Consequently, the radiosensitivity in the asynchronous phase (general cell phase) can be nearly determined by that of the G_1_/S phase. Considering the improvements in the uptake fractions in the G_1_/S phase, the use of PVA is an effective way to diminish the heterogeneous uptake and improve the curative effects of BNCT. In addition, the cell cycle-specific anticancer agents enabling the G_2_/M accumulation are expected to improve the RBE values of BNCT. The estimate of improving the RBE by cell cycle is depicted in Fig. S15 of Supplementary Material B.

## Discussion

To the best of our knowledge, this is the first study to demonstrate the cell cycle dependency of intracellular BPA and BPA-PVA concentrations and the biological effects of BNCT. By focusing on the cell cycle dependence on the ^10^B uptake fractions (Fig. 3k), the fraction ratios of the S/G_2_/M phase to the G_1_/S phase increased as the administered BPA densities increased (2.17 for 6 ppm, 1.330 for 20 ppm, and 1.215 for 60 ppm). This indicates that the difference would be more pronounced at lower BPA blood concentrations. In general, for successful BNCT, BPA blood concentrations of at least 20 ppm within tumors are needed^45^. Our findings indicate that the heterogeneities of intracellular ^10^B densities under the > tens of ppm can be suppressed, compared with those in the case of low BPA densities (i.e., 6 and 20 ppm). Regarding the reliability of our experimental results, Yoshida et al*.* investigated the cell cycle dependence of the sodium borocaptate (BSH) and the BPA, and found a similar trend of higher uptake in the G_2_/M than in the G_0_/G_1_ phase^14^. Considering all the above factors, our experimental system can be considered to function well scientifically. The difference in the uptake was found to be more prominent in the case of BPA^14^, which might be intrinsically related to the L-type amino acid transporter (i.e., LAT1)^46^. Meanwhile, it has been reported that the PVA-BPA can be locally taken up in the endo-/lysosomes through LAT1-mediated endocytosis^7,47^. However, there is no biological report investigating the cell cycle dependences of LAT1 and LAT1-mediated endocytosis. Further explorations of these boron uptake mechanisms are mandatory in future BNCT development.

As one of the technical limitations of this study, the possible biological effects were evaluated using the IMK model prediction without any experiments of the clonogenic survival assay (generally performed in vitro to determine radiosensitivity). This is because temperature control under the irradiation setting is technically difficult because of the long exposure time (≤ 48 h). To overcome this limitation, fixed samples were prepared, and the high LET ion tracks were directly measured using the CR-39 detector. Model verification was conducted in comparison with the experimental values in the V79 and HSG cell lines in previous reports^48–50^. In this regard, the estimation accuracy of the IMK model for HeLa cells must be evaluated. Furthermore, a preliminary verification of the IMK model was performed in HeLa cells (in an asynchronous phase) for various ions. The IMK model successfully reproduced the experimental RBE^51–57^ of the HeLa cells (including the Particle Irradiation Data Ensemble database^57^) for various LET radiation types (Fig. S16 of Supplementary Material B). Furthermore, we compared the relative radiosensitivity of BNCT to neutron irradiation estimated based on the BPA concentrations measured in this study (Fig. 3f and g) to the experimental values reported by Davis et al., which was measured by 92% enriched ^10^B boric acid^58^. An additional test found that the model prediction considering the saturation of BPA uptake and the lower uptake in HeLa cells reasonably has good agreement with the experimental data^58^ (Fig. S17 of Supplementary Material B).

In general, the order of cell cycle-dependent radiosensitivity is G_2_/M > G_1_ > S^59^. The difference is considered intrinsically related to the DNA content and damage repair system^60^. The doubled DNA contents are contained in the G_2_ phase compared with the G_1_ phase, which enhances the initial yield of DNA double-strand breaks (DSBs)^61^, which can lead to cell death with a certain probability^62^. Meanwhile, the two types of repair processes are nonhomologous end joining and homologous recombination (HR)^63^. A more accurate repair process named HR becomes more important in the S phase, contributing to reducing radiosensitivity in the S phase^64^. These processes can modify the model parameter set (${\alpha}_{{0}\star }$, ${\beta}_{\star }$) even in the same type of cell line^60^, leading to the modification of RBE values by the cell cycle phase (Fig. 5a and b). In AB-BNCT irradiation, a single irradiation with a high dose was adopted as a clinical dose–delivery plan^65^. Considering the lower value of ${\beta}_{\star }$ in the G_1_/S phase than that in the S/G_2_/M phase, the G_1_/S phase exhibits resistance to radiation during the cell cycle phase. Based on this, the increment of boron uptake in the G_1_/S phase to the level of the S/G_2_/M phase (Fig. 4c and e) is expected to improve the curative effects while minimizing the energy absorbed in normal tissues because the irradiation time (incident neutron number) required to achieve the same biological effect can be reduced.

As cell cycle progression and checkpoints are involved in the mechanism of action of several anticancer agents, most outcomes of radiotherapy with these agents depend on the cell cycle. Our findings support that it is reasonable to inhibit cell divisions with chemotherapy, thereby preserving the ^10^B uptake of the S/G_2_/M phases for BNCT. Further elucidation of the ^10^B uptake of each phase of the cell cycle, in a more extensive range of cancer cell models, and its mechanism would yield novel insights into the improvement of BNCT in combination with cell cycle-specific anticancer agents. Alternatively, the possibility of using a concomitant agent that equalizes ^10^B-BPA uptake by the cell cycle phase, such as the PVA used in this study, would be promising.

## Conclusions

**Figure 6:**
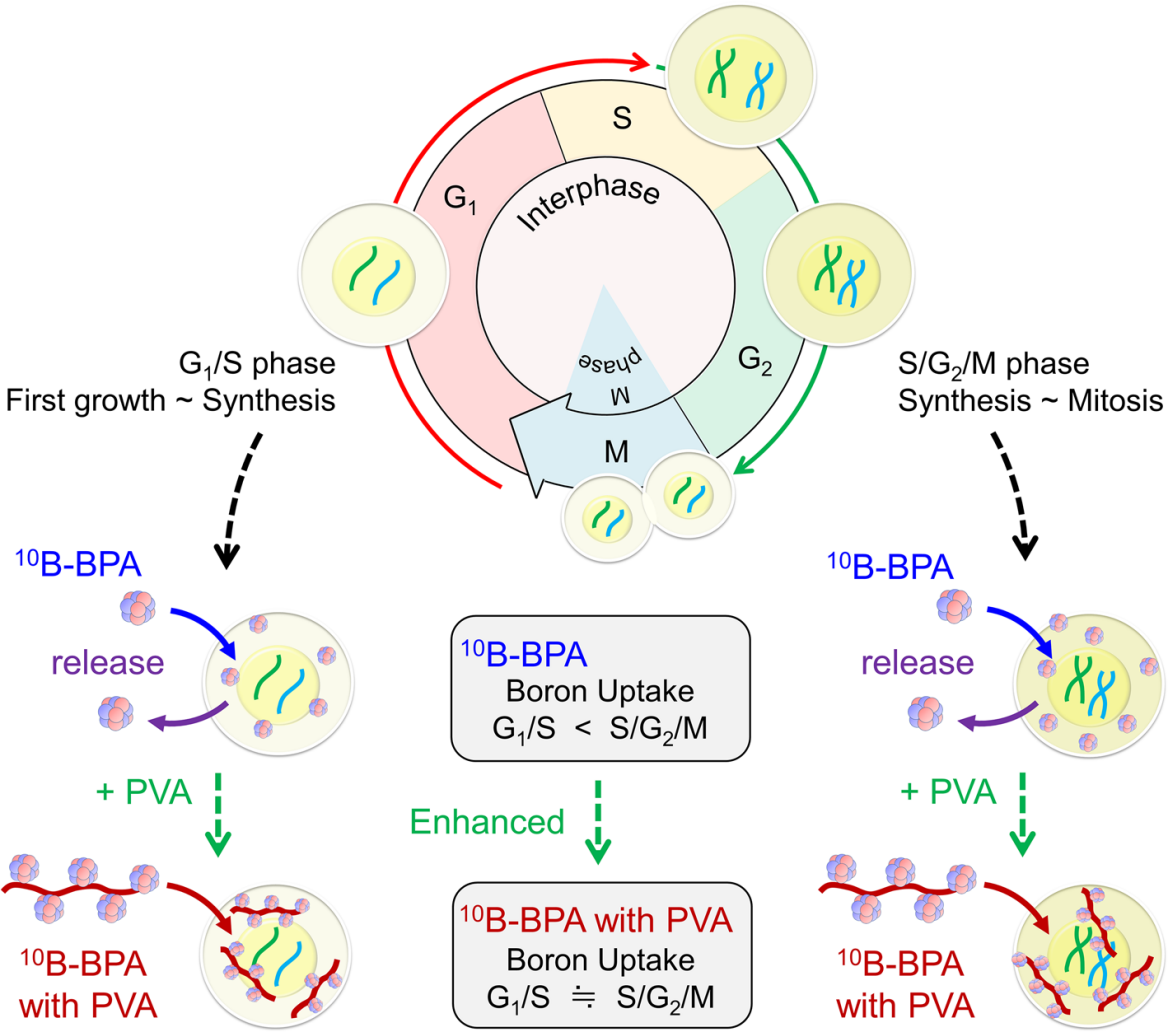
Overview of cell cycle dependent boron uptake. We investigated the boron concentration within cells in the G_1_/S phase (red allow) and that in the S/G_2_/M phase (green allow) in the cell cycle, as shown in the upper part of the figure. In the case of BPA administration, BPA can be taken up in cells, but boron cannot be fully taken up (leading to the uptake saturation) due to release (central figure). In addition, boron uptake in the S/G_2_/M phase is significantly higher than in the G_1_/S phase. In the case of PVA-BPA administration, boron uptake can be enhanced, and there is no cell cycle dependence, which is expected to enhance cell killing in the radioresistant phase of the G_1_/S phase.

By adopting an interdisciplinary approach with the CR-39 plastic detector, FUCCI probe, PHITS, and IMK model, we successfully quantified the cell cycle dependence of intracellular ^10^B concentration (temporospatially heterogeneous boron uptake) and determined the possible biological effects for AB-BNCT. Figure 6 shows a summary of the cells’ boron uptake when administering BPA and PVA-BPA. The experimental results showed that the ^10^B concentration in the S/G_2_/M phase was significantly higher than that in the G_1_/S phase, whereas the concentration was saturated at 200 ppm of administered boron concentration or more because of biological equilibrium. Notably, the cell cycle dependence of ^10^B concentration and uptake fraction can be diminished by PVA-BPA. Considering that the G_1_/S phase demonstrated higher radioresistance than the G_2_/M phase, the improvement of boron uptake and cancelation of cell cycle dependence by PVA enhances the cell killing effects of BNCT irradiation. These findings may contribute to the robustness of BNCT when combined with cell cycle-specific anticancer agents in the future.

### Supplementary Information

Supplementary Information 1.

Supplementary Information 2.

## Data Availability

The data supporting the findings of this study are available within the article and its supplementary materials.
