# Supplementary material for: Cell-cycle dependence on the biological effects of boron neutron capture therapy and its modification by polyvinyl alcohol
Source: Sci Rep. 2024 Jul 19;14:16696. doi: 10.1038/s41598-024-67041-6 (PMC11271528; doi:10.1038/s41598-024-67041-6)
Supplement: Supplementary file 1 — Supplementary Information 1. [file 41598_2024_67041_MOESM1_ESM.pdf]

# *Experimental setup and preliminary results under the accelerator-based neutron irradiation*

*Supplementary Material A of "Cell-cycle dependence on the biological effects of boron neutron capture therapy and its modification by polyvinyl alcohol"*

This supplementary file includes the experimental setups and preliminary results under the accelerator-based (AB) neutron irradiation to measure the concentration of BPA within HeLa and HeLa-fluorescent ubiquitination-based cell-cycle indicator (FUCCI) cells. The HeLa-FUCCI cell line is presented by HeLa cells expressing FUCCI (Sakaue-Sawano *et al*. 2008, 2014), which enables the visualization of each phase of the cell-cycle via fluorescent microscopy as shown in Figs. 1a, 3a, and 4a in the main paper. To measure the BPA concentrations within cells, various preliminary tests were evaluated. Herein, we present (1) the radiation fields of the AB neutron based on a 3.0-MeV proton accelerator on the Li target, (2) the detection efficiency of ions by the CR-39 plastic detector, (3) the cell-cycle condition of HeLa and HeLa-FUCCI cells, and (4) raw data of etch pits within cells detected by the CR-39 detector.

First, we evaluated the fluxes of AB neutrons, recoiled protons, and prompt γ-rays in our experiment using the Particle and Heavy Ion Transport code System (PHITS) (Sato *et al*. 2018). AB neutrons were irradiated at the Fast Neutron Laboratory at Tohoku University (Baba *et al.* 1996). The experimental setup at the Fast Neutron Laboratory at Tohoku University is illustrated in Fig. S1a. The thermal neutron flux was measured with a scintillator using optical fiber (SOF) (Ishikawa *et al*. 2015) at the outside of the polyethylene block located the farthest from the Li target (neutron source). Then, the experimental geometry was reproduced, and the fluxes of the neutrons, protons, and γ-rays were calculated using the PHITS code.

Figure S1b depicts the geometry considered in the PHITS code from different view angles in Fig. 2a, which was illustrated using the PHIG-3D software (Ohnishi 2021). The energy spectra and depth dependence of the fluxes are summarized in Fig. 2b and 2c. The neutron spectra used in this simulation were verified using the BF3 and CR-39 detectors in our previous paper (Matsuya *et al.* 2022). Based on the measurement using the SOF detector and PHITS simulation, the numbers of protons, α-particles, and Li ions per μm$^2$ at the surface of the cell layer on CR-39 were calculated [Fig. 2c], and the BPA concentration within the HeLa cells was quantified from the number of etch pits per μm$^2$. CR-39 enables the detection of high-LET ions such as a part of recoiled protons and heavy ions (α-particles and Li ions). Figure S2 shows the spatial distribution of the radiation tracks (S2a and S2d for neutrons, S2b for γ-rays, S2c and S2e for protons, and S2f for α-particles and Li ions) calculated using the PHITS code. When depicting the 2D distribution, the electron gamma shower mode (Hirayama *et al*. 2005), INCL (Boudard *et al.*

2013) and event generator mode (Ogawa *et al*. 2014) were used. In the same manner as the main paper, the cutoff energies, except for neutrons, were set to 1.0 keV, whereas those for neutrons were set to $1.0 \times 10^{-4}$ eV.

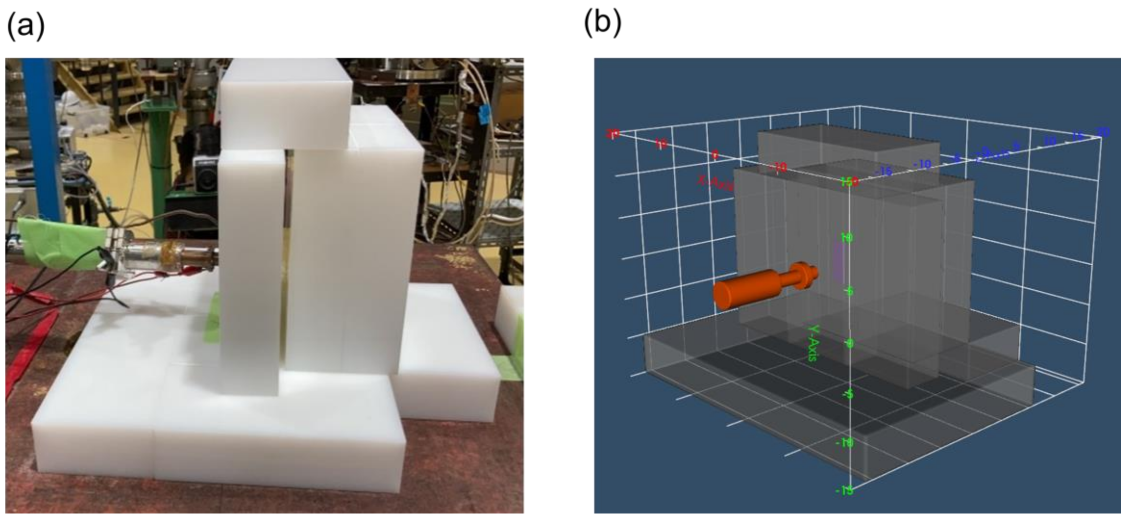


**Figure S1. Experimental geometry for irradiating neutrons**: (a) is the picture of the Fast Neutron Laboratory at Tohoku University, and (b) is the simulation geometry considered in the PHITS code from the different view from Fig. 2.

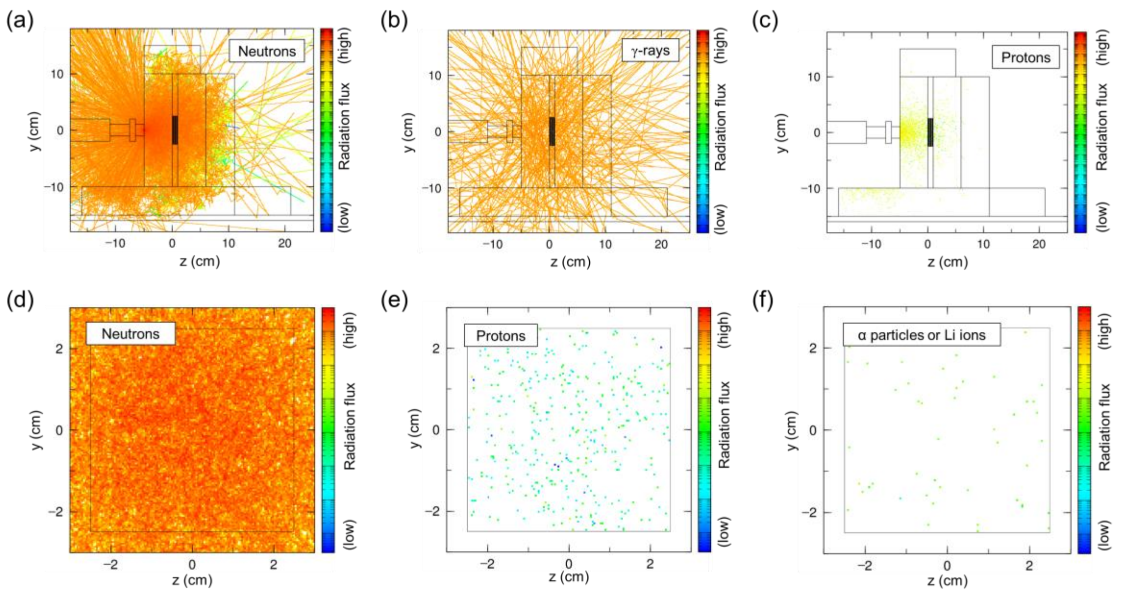


**Figure S2. Radiation flux (trajectories) calculated by the PHITS code**: (a-c) for the fluxes of neutrons, photons, and protons in the experimental geometry (x-z surface), (d-f) for the flux of neutrons, protons, and α-particles and Li ions at the surface of the first CR-39 detector (x-y surface).

Second, when quantitatively evaluating the BPA concentrations from the number of chemical etch pits of the CR-39 detector, the detection efficiencies of the CR-39 detector (BARYOTRCK, Fukuvi Chemical Ltd.) must be determined for the recoiled protons and ions

generated by $^{10}B(n,\alpha)^{7}Li$ reactions, as shown in Fig. S3a. To obtain the efficiencies, we prepared the dried cell samples with and without BPA administrations. The administered BAP concentration was 6000 ppm. Using the concentrations, chemical etchings were also performed using the potassium–hydroxide–ethanol–water solution with ethanol concentrations of 15wt.% (so-called PEW-15) and those by 0wt.% (PEW-0) solution (Ogawara *et al.* 2020). The treatment time and temperature for the PEW-15 and PEW-0 were 50°C for 280 min and 70°C for 1613 min, respectively. Consequently, as shown in Fig. S3b and S3c, the efficiencies of α-particles and Li ions for PEW-0 and PEW-15 were 92.9% ± 4.6% and 62.9% ± 2.8%, respectively, whereas those of recoiled protons for PEW-0 and PEW-15 were 57.8% ± 0.9% and 12.2% ± 0.2%, respectively. These results indicate that the CR-39 detector with the PEW-15 allows the 57.8% detection of the $^{10}B(n,\alpha)^{7}Li$ reactions. The 12.2% of recoiled protons can be also detected as a noise when evaluating the number of the $^{10}B(n,\alpha)^{7}Li$ reactions. Considering these, the number of recoiled protons (each pits) was subtracted from the total that of etch pits and that of $^{10}B(n,\alpha)^{7}Li$ reactions was corrected based on the detection efficiency of 57.8% ± 0.9%.

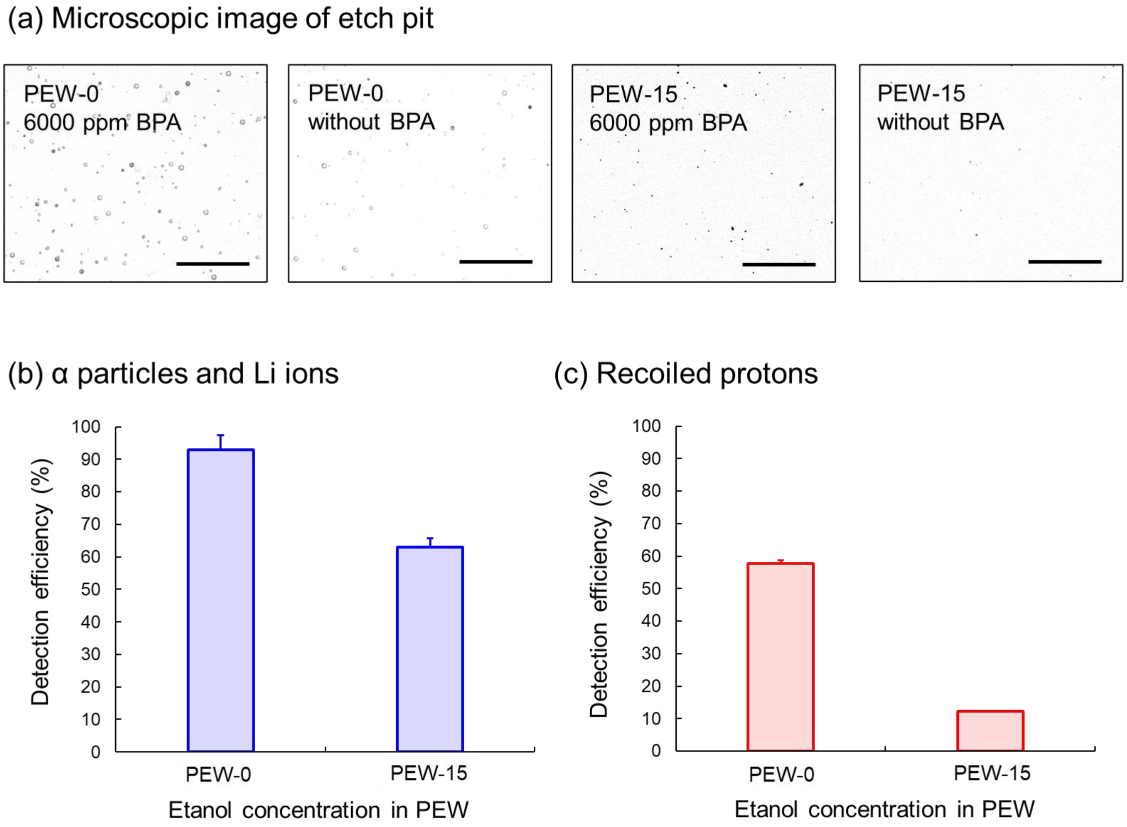


**Figure S3. Chemical etch pits and the detection efficiencies**: (a) is the microscopic image of etch pits, (b) is the detection efficiencies of α-particles and Li ions for PEW-0 and PEW-15 solutions, (c) is the efficiencies of recoiled protons for PEW-0 and PEW-15 solutions. From these results, we confirmed that the efficiencies of α-particles and Li ions for PEW-0 and PEW-15 were 92.9% ± 4.6% and 62.9% ± 2.8%, respectively, while those of recoiled protons for PEW-0 and PEW-15 were 57.8% ± 0.9% and 12.2% ± 0.2%, respectively.

Third, before measuring the nuclear reaction generated within cells, the cell-cycle distributions (i.e., $G_1/S$ and $S/G_2/M$ phases) of the HeLa and HeLa-FUCCI cell lines used in this experiment were evaluated. The distribution of HeLa-FUCCI cells was quantified using the color of the FUCCI system (i.e., red, $G_1$ phase; yellow, early S phase; green, $S/G_2/M$ phase). Meanwhile, that of the HeLa cells was measured by propidium iodide (PI) using an Attune acoustic focusing flow cytometer (Applied Biosystems by Life Technologies). As the experimental protocol, the HeLa cells were fixed with 70% ethanol and then kept at 4°C for at least 2 h. After centrifugation, the cells were resuspended in 1 mL of phosphate-buffered saline (PBS)(−). After centrifugation, the DNA was stained with 0.5 mL of FxCycleTM PI/RNase staining solution (Life Technologies) including 0.2% v/v triton X for 15 min in the dark at room temperature. The measured cell-cycle distributions are shown in Fig. S4, where Fig. S4a and S4b are those of the HeLa-FUCCI cells, and Fig. S4c and S4d are those of the HeLa cells. As shown in Fig. S4, the distributions of the HeLa-FUCCI cells were nearly the same as those of the HeLa cells, indicating that the FUCCI factor and BPA administration do not affect cell growth without any cell toxicities.

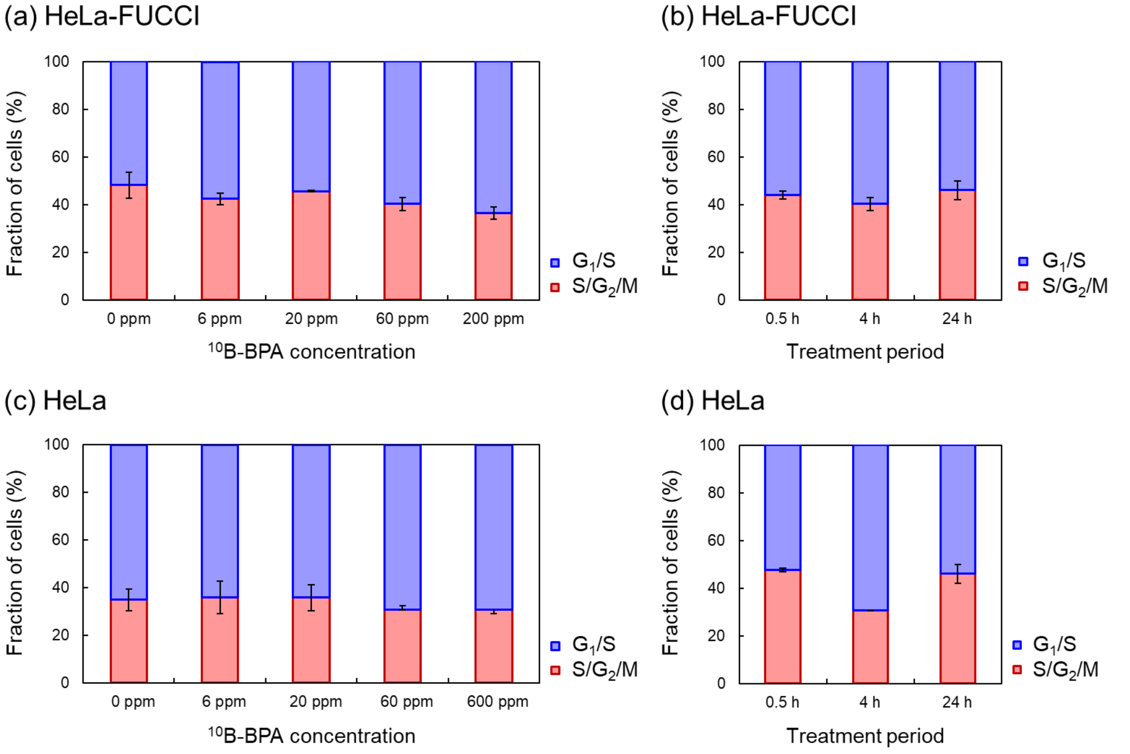


**Figure S4. Cell-cycle distributions of HeLa and HeLa-FUCCI cell lines**: (a) and (b) for the HeLa-FUCCI cells, and (c) and (d) for the HeLa cells. The fraction of cell-cycle distribution for HeLa-FUCCI was measured by using the FUCCI system (red and yellow, $G_1$ phase; green, $S/G_2/M$ phase). Meanwhile, the fractions for HeLa cells were measured by using propidium iodide (PI) and the Attune acoustic focusing flow cytometer (Applied Biosystems by Life Technologies TM).

Fourth, the raw data of etch pits within cells detected by the CR-39 detector before converting the BPA concentration from the number of etch pits were presented. When counting the number of etch pits within the cells, the cross section of the cells was also measured in μm$^2$. The number of etch pits was calculated per μm$^2$, and the BPA was converted from the number by comparing the fluence of heavy ions (such as α-particles and Li ions) calculated using the PHITS code. Figure S5 shows the number of etch pits per μm$^2$ before the correlations, where Fig. S5a and S5c present the numbers of the HeLa cells and Fig. S5b and S5d present those of the HeLa-FUCCI cells. The background (i.e., recoiled protons) detected in the outside regions of the cells varies for each condition because the depth of the CR-39 detectors from the AB-neutron source (Li target) varies. The depth dependence of the proton fluence is shown in Fig. 2c in the main paper. By using the raw data shown in Fig. S5, we obtained the BPA concentrations within the cells.

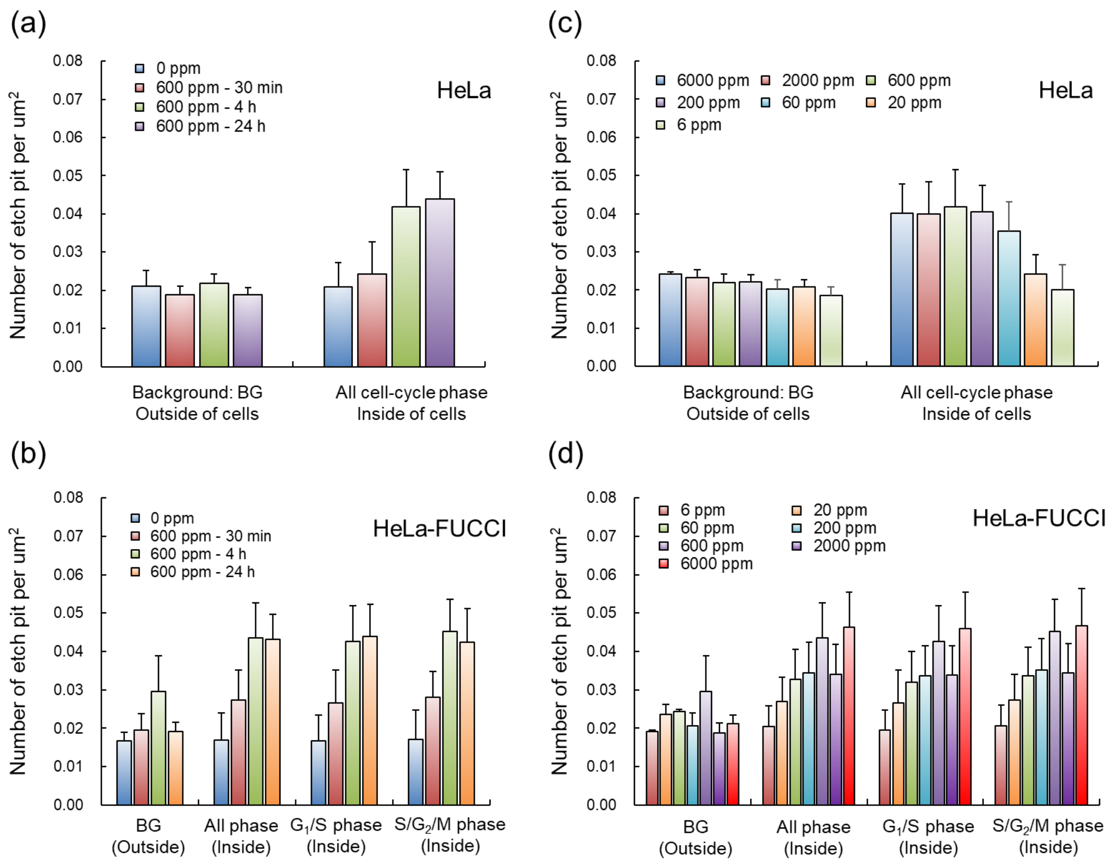


**Figure S5. Number of etch pits per μm$^2$ after the BPA administration**: (a) and (b) show the dependences of etch pits after administering 600 ppm BPA on the treatment period for HeLa and HeLa-FUCCI cell lines, respectively, and (c) and (d) show the dependences of etch pits on administered BPA concentration for HeLa and HeLa-FUCCI cell lines, respectively. BG represents the background by the recoiled protons detected outside the cells.

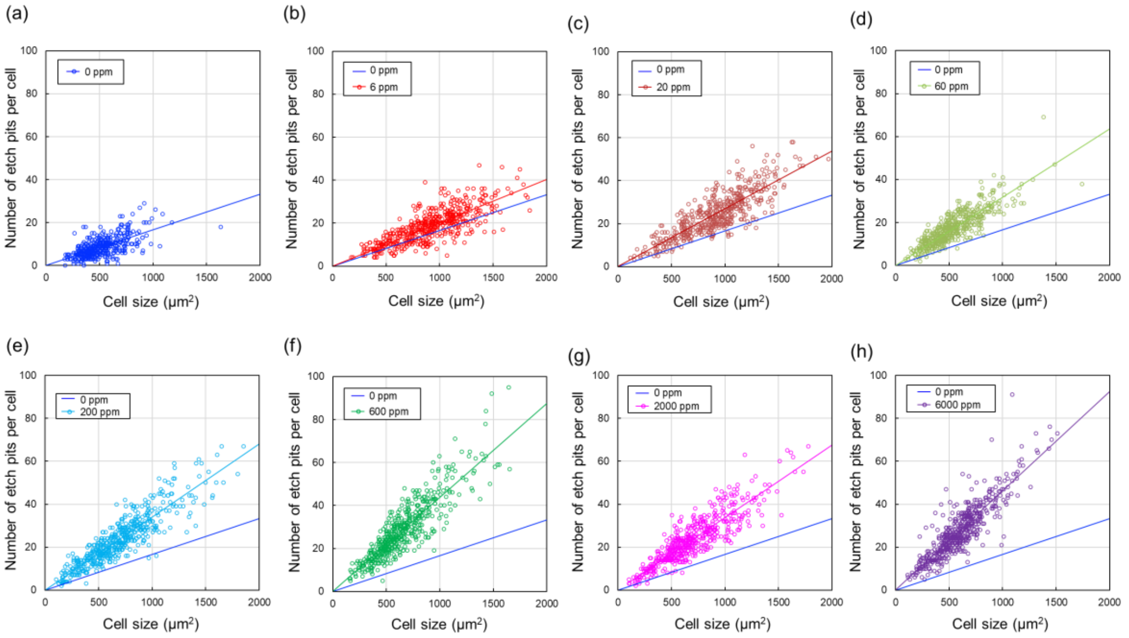


**Figure S6. Relationship between cell size (cross section) and etch pits per cell after the BPA administration**: (a) 0 ppm (b) 6 ppm, (c) 20 ppm, (d) 60 ppm, (e) 200 ppm, (f) 600 ppm, (g) 2000 ppm, (h) 6000 ppm. From this relationship, it was confirmed that the number of etch pits per μm$^2$ for each BPA density is constant, and, boron concentration can be obtained from etch pits per μm$^2$ compared to the PHITS code (theoretical radiation flux).

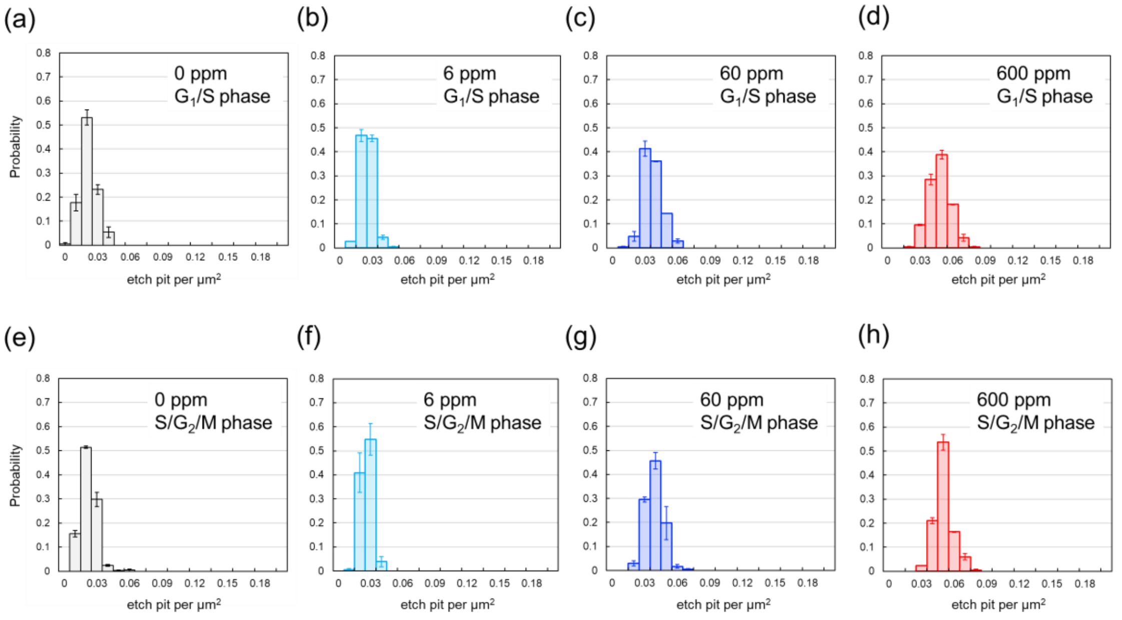


**Figure S7. Histogram of etch pits per μm$^2$ for $G_1$/S phase and S/$G_2$/M phase after BPA administration**: (a) 0 ppm for $G_1$/S phase (b) 6 ppm for $G_1$/S phase, (c) 60 ppm for $G_1$/S phase, (d) 600 ppm for $G_1$/S phase, (e) 0 ppm for S/$G_2$/M phase (f) 6 ppm for S/$G_2$/M phase, (g) 60 ppm for S/$G_2$/M phase, (h) 600 ppm for S/$G_2$/M phase. The increase in the S/$G_2$/M phase can be observed compared to those in the $G_1$/S phase.

As the experimental data of etch pits, the relationship between the cell size (in μ$m^2$) and the number of etch pits in a cell was also obtained, which is shown in Fig. S6. The number of etch pits in a cell is proportional to the cell size. As discussed in the main text, the slopes shown in Fig. S6 were saturated at approximately ≥200 ppm. The tendency can be seen in Fig. 3f–3j. From these results, there is a limit to the BPA uptake in the HeLa cells as well as the HeLa-FUCCI cells. Figure S7 shows the distribution of the number of etch pits per μ$m^2$ in the HeLa-FUCCI cells, in which Fig. S6a–S6d presents the $G_1$/S phase and Fig. S6e– S6h illustrates the S/$G_2$/M phase. Focusing on the mean cellular BPA concentrations [Fig. 3j and 3k], the BPA uptake in the S/$G_2$/M phase is higher than that in the $G_1$/S phase. As for the distribution of individual cells, the same tendency of the high uptake in the S/$G_2$/M phase can be also observed, as shown in Fig. S6e– S6h. From these results [Fig. 3j, 3k, Fig. S6e–S6h], we confirmed that the heterogeneous BPA concentrations within cells are predominantly attributed to the difference of the $^{10}$B uptake between the $G_1$/S and S/$G_2$/M phases.

Finally, the preliminary results of the PVA-BPA effects on cell growth and cell toxicity are presented. In the main text, the cell experiments were performed using the PVA at final concentrations of 0.2% and 0.02%. In the preliminary test, we tried to culture the HeLa cells on the CR-39 cells in the case of 2% PVA. Figure S8 depicts the microscopic images of the use of PVA-BPA on HeLa cells, where (A) is 2% PVA + 20-ppm BPA, (B) is 0.2% PVA + 20-ppm BPA, (C) is 0.02% PVA + 20-ppm BPA, and (D) is 0% PVA + 20-ppm BPA. As shown in these microscopic images, the 2% PVA adhered very tightly to the cells and cannot be removed by washing with PBS(−). Under this condition, detecting the BPA concentration taken up within the cells is difficult. The pH of the cell culture medium (RPMI-1640 containing 10% fetal bovine serum [FBS] and 1% p/s), including the PVA-BPA at various final concentrations, was also checked, as shown in Fig. S9. The result revealed no significant differences in the pH for the medium including PVA-BPA compared with that for the medium without PVA and the PBS(−). Figure S10 compares the cell-cycle distributions for 0-ppm BPA (control group), 20-ppm BPA, 2% PVA + 0-ppm BPA, and 2% PVA + 20-ppm BPA. Compared with the control group, PVA had no huge effects on cell-cycle distributions 4 h after administration. Considering comprehensively, we used 0.2% PVA as a maximal concentration in the experiment.

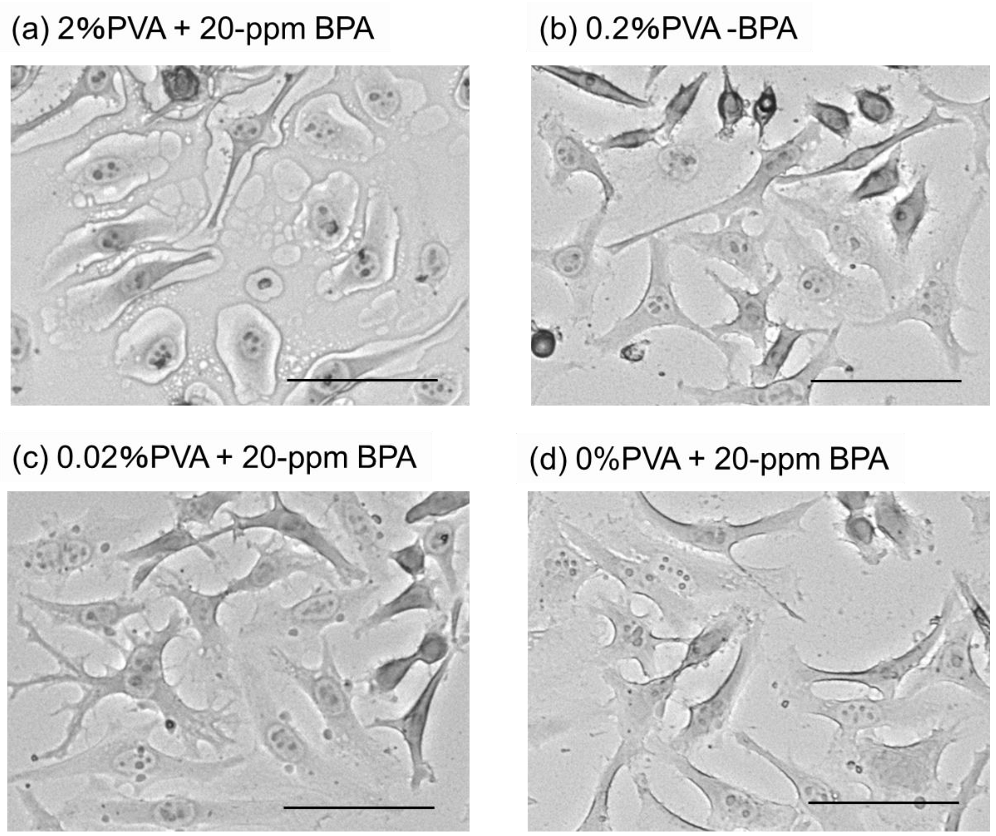


**Figure S8. Microscopic image for the use of PVA-BPA on HeLa cells**: (a) 2% PVA + 20-ppm BPA, (b) 0.2% PVA + 20-ppm BPA, (c) 0.02% PVA + 20-ppm BPA, (d) 0% PVA + 20-ppm BPA. From these microscopic images, we decided to use the 0.2% PVA as a maximal concentration in the experiment. In Fig. S8, the scale bar represents 100 μm.

.

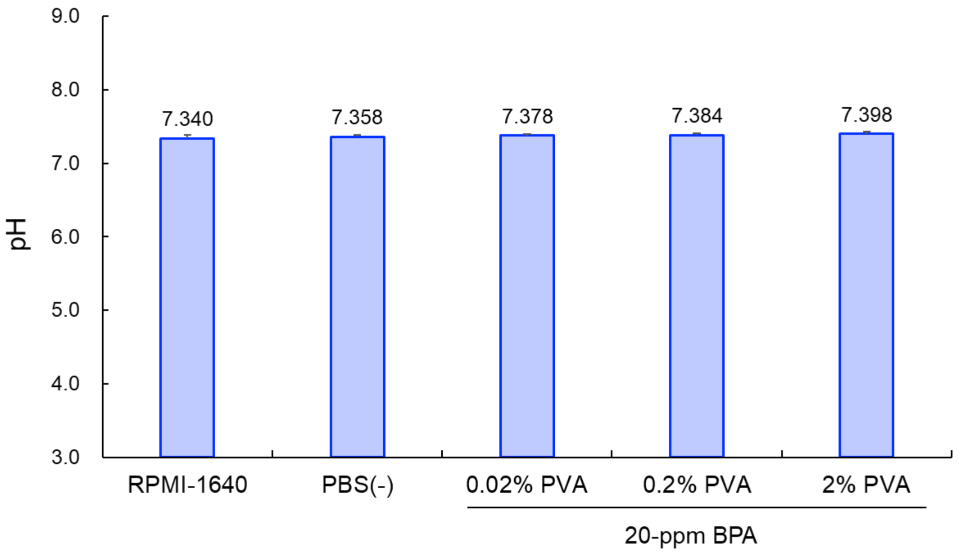


**Figure S9. Measured the pH of each PVA concentration**. The PVA-BPA was added in the cell culture medium (RPMI-1640, Thermo Fisher Scientific Inc. Tokyo, Japan) with 10% fetal bovine serum (FBS, Nichirei Bioscience Inc., Tokyo, Japan) and 1% penicillin/streptomycin (p/s). Note that the legend of "RPMI-1640" in the horizontal axis means the medium including 10% FBS and 1% p/s. The pH of the PBS(−) was also measured as a control. There were no dramatic differences in the pH between them.

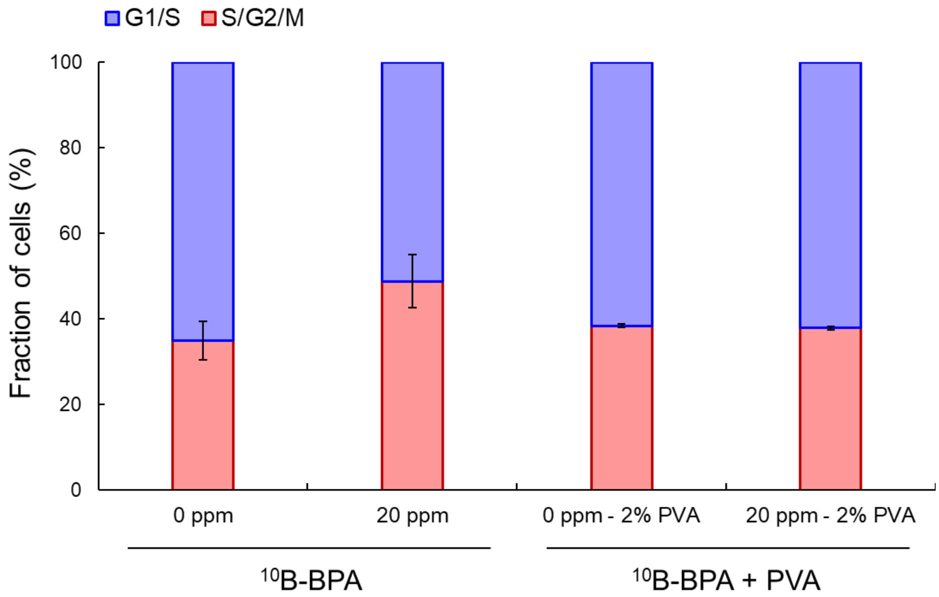


**Figure S10. Cell-cycle distributions 4h after administering PVA**. The concentration of the PVA and the BPA were 2% and 20 ppm, respectively. We added the PVA-BPA in the RPMI-1640 medium containing 10% FBS and 1% p/s and administered the medium including PVA-BPA to the HeLa cells. As a result, there were no dramatic differences in cell-cycle distribution between them.

## References

Ando R, Sakaue-Sawano A, Shoda K, Miyawaki A 2020 Two new coral fluorescent proteins of distinct colors for sharp visualization of cell-cycle progression. *bioRxiv* 2020.03.30.015156.

Baba M, Takada M, Iwasaki T, Matsuyama S, Nakamura T, Ohguchi H, Nakao T, Sanami T, Hirakawa N 1996 Development of monoenergetic neutron calibration fields between 8 keV and 15 MeV. *Nucl. Instrum. Methods Phys. Res. Sect. A* 376, 115–123.

Boudard A, Cugnon J, David J-C, Leray S, Mancusi D 2013 New potentialities of the Liège intranuclear cascade model for reactions induced by nucleons and light charged particles. *Phys. Rev. C* 87, 014606.

Hirayama H, Namito Y, Bielajew AF, Wilderman SJ, Nelson WR 2005 The EGS5 Code System; Office of Scientific and Technical Information (OSTI): Oak Ridge, TN.

Ishikawa M, Tanaka K, Endo S, Hoshi M 2015 Application of an ultraminiature thermal neutron monitor for irradiation field study of accelerator-based neutron capture therapy. *J. Radiat. Res.* 56, 391–396.

Ogawa T, Sato T, Hashimoto S, Niita K 2014 Development of a reaction ejectile sampling algorithm to recover kinematic correlations from inclusive cross-section data in Monte-Carlo particle transport simulations. *Nucl. Instrum. Methods Phys. Res. Sect. A* 763, 575–590.

Ogawara R, Kusumoto T, Konishi T, Hamano T, Kodaira S. Detection of alpha and $^{7}$Li particles from $^{10}$B(n, α)$^{7}$Li reactions using a combination of CR-39 nuclear track detector and

potassium hydroxide-ethanol-water solution in accelerator-based neutron fields 2020 *Nucl. Instrum. Methods Phys. Res., Sect. B* 467, 9–12.

Ohnish, S 2021 Gxsview: Geometry and cross section viewer for calculating radiation transport. *SoftwareX*, 14, 100681.

Sakaue-Sawano A, Kurokawa H, Morimura T, Hanyu A, Hama H, Osawa H, Kashiwagi S, Fukami K, Miyata T, Miyoshi H, Imamura T, Ogawa M, Masai H, Miyawaki A 2008 Visualizing Spatiotemporal Dynamics of Multicellular Cell-Cycle Progression, *Cell* 132, 487–498.

Sakaue-Sawano A, Miyawaki A 2014 Visualizing Spatiotemporal Dynamics of Multicellular Cell-Cycle Progressions with Fucci Technology. *Cold Spring Harb. Protoc*. (5), pdb.prot080408

Sato T, Iwamoto Y, Hashimoto S, Ogawa T, Furuta T, Abe S Kai T, Tsai P-E, Matsuda N, Iwase H, Shigyo N, Sihver L, Niita K 2018 Features of Particle and Heavy Ion Transport code System (PHITS) version 3.02. *J. Nucl. Sci. Technol*. 55(5–6), 684–690.

Matsuya Y, Kusumoto T, Yachi Y, Hirata Y, Miwa M, Ishikawa M, Date H, Iwamoto Y, Matsuyama S, Fukunaga H 2022 Features of accelerator-based neutron source for boron neutron capture therapy calculated by particle and heavy ion transport code system (PHITS). *AIP Adv.* 12, 025013.
