# Supplementary material for: Cell-cycle dependence on the biological effects of boron neutron capture therapy and its modification by polyvinyl alcohol
Source: Sci Rep. 2024 Jul 19;14:16696. doi: 10.1038/s41598-024-67041-6 (PMC11271528; doi:10.1038/s41598-024-67041-6)
Supplement: Supplementary file 2 — Supplementary Information 2. [file 41598_2024_67041_MOESM2_ESM.pdf]

Benchmark test for the estimation of biological effects using the integrated microdosimetric–kinetic model

Supplementary Material B of "Cell-cycle dependence on the biological effects of boron neutron capture therapy and its modification by polyvinyl alcohol"

In this supplementary file, we present the preliminary results of the benchmark test for the estimates of the biological effects of the HeLa (HeLa-FUCCI) cell lines (i.e., surviving fraction and relative biological effects [RBE]) using the integrated microdosimetric–kinetic (IMK) model (Matsuya *et al*. 2020, 2023). The IMK model, a biophysical model, enables the prediction of surviving fractions. The IMK model was developed based on the MK model that considers microdosimetry (ICRU 1983, Hawkins 1996) and the overkill effects that are induced by high-linear-energy-transfer (LET) radiations (Kase *et al*. 2013). To date, the original MK model has been applied to calculate the RBE-weighted dose for particle therapies (Inaniwa *et al*. 2013, Takada *et al*. 2020). In general, the MK model has been verified in the comparison of the experimental in vitro data in the V79 (Chinese Hamster lung fibroblast) and human salivary gland adenocarcinoma (HSG) cell lines (Kase *et al*. 2013, Inaniwa *et al*. 2013); however, such verification has not been performed for the HeLa (HeLa-FUCCI) cell lines. Herein, we present the calculated data not shown in the main manuscript and the benchmark results of the IMK model applied to HeLa cell line data.

When predicting the dose–response curve of the cell surviving fraction, the IMK model must fit at least one experimental dataset after radiation, i.e., photon irradiation. In the main manuscript, the IMK model [Eq. (1)] was fitted to the experimental dose responses of the HeLa-FUCCI cells in the G_1/S and S/G_2/M phases (Seino *et al*. 2023) using a fitting program, such as a Markov chain Monte Carlo (MCMC) simulation (Matsuya *et al*. 2017), which is a Bayesian estimation method that enables the sampling of the probability distribution of interest (IMK model parameters in this study). The details of the MCMC simulation are summarized in the main manuscript (Materials and Methods: Estimate of RBE based on the MK model and Particle and Heavy Ion Transport code System (PHITS)). The mean and standard deviation of the cell cycle-specific model parameters ($\alpha_{0\star}$ [Gy^{-1}], $\beta_\star$ [Gy^{-2}]) are listed in Table 1 in the main manuscript. The posterior distributions of $\alpha_\star$ (= $\alpha_{0\star} + \gamma\beta_\star$) and $\beta_\star$ after the MCMC simulation for the G_1/S and S/G_2/M phases are depicted in Fig. S11a and S11b, respectively. As illustrated in Fig. S11, using the MCMC simulation, this work enables the evaluation of the uncertainties of the IMK model parameters ($\alpha_{0\star}$ [Gy^{-1}], $\beta_\star$ [Gy^{-2}]). Note that $\star$ represents certain cell cycle phases (i.e., G_1/S or S/G_2/M).

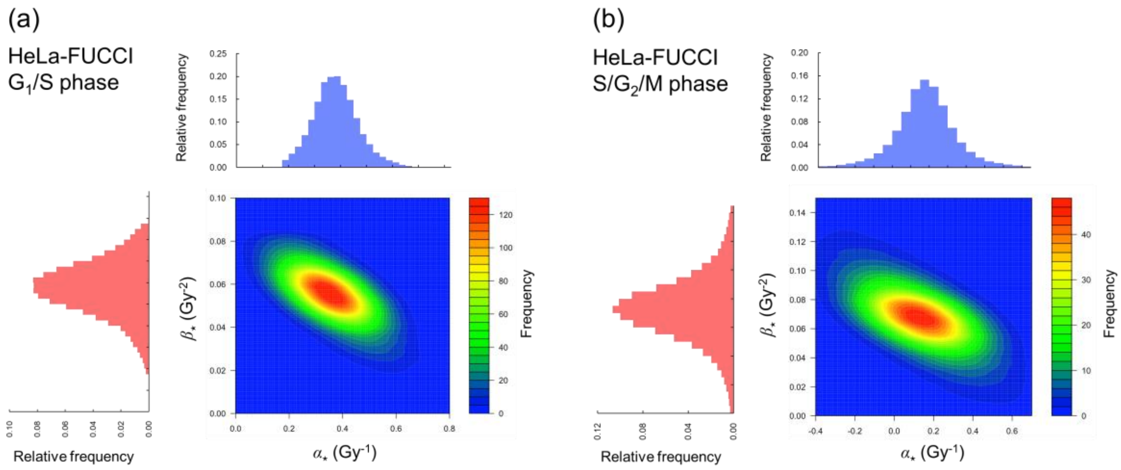

Figure S11. Model parameters sampled using the MCMC simulation: (a) is the posterior distribution of the model parameters for the HeLa cells in the G_1/S phase, and (b) is that for the cells in the S/G_2/M phase. Note that we assumed the uniform distributions for $\alpha_{0\star}$ and $\beta_\star$ as a prior distribution.

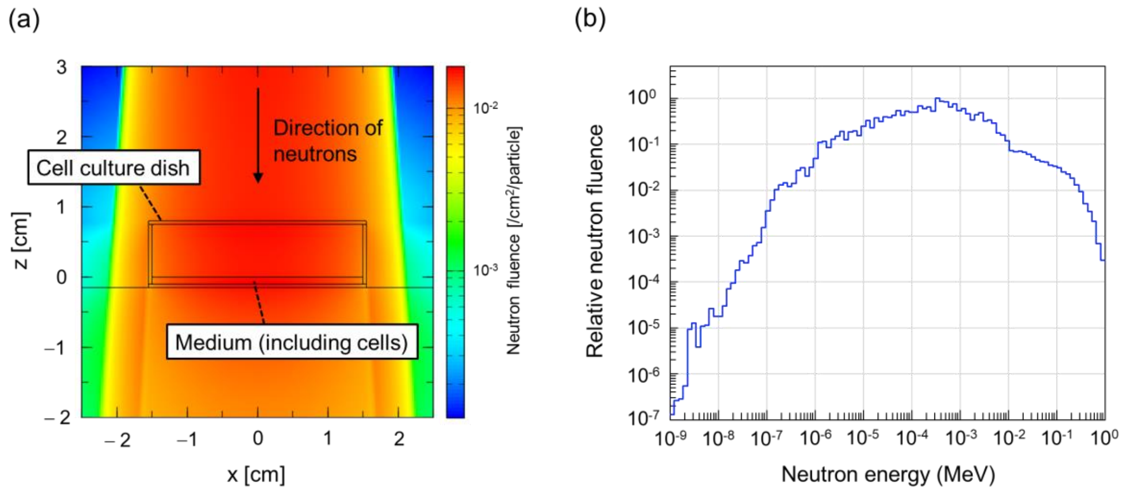

Figure S12. Simulation of accelerator-based neutrons for estimating biological effects: (a) is the simulation geometry considering *in vitro* experiments. (b) is the energy spectra of AB-neutrons used in the PHITS simulation. The neutron energy spectrum was sampled using the *t-cross* tally which enables us to obtain the fluences in any specified surface in the PHITS code.

In the main text, the dose–response curve of the surviving fraction was estimated after the AB-neutron and BNCT irradiation (Fig. 5 in the main text). To estimate the curves of the AB-neutron irradiation, the microdosimetric quantities, i.e., lineal energy spectra and y^* value (ICRU 1983, Kase *et al*. 2013) must be evaluated. As written in the main text, the neutron field at Tohoku University, which was used for this experiment, is not a BNCT facility. Considering this, when evaluating the biological effects, the clinical AB-neutron spectra in the BNCT facility based on a 2.5 MeV proton accelerator on the Li target reported in the literature were used (Fantidis 2018). The y^* values for AB-neutron fields were calculated using a general-purpose Monte Carlo code for radiation transport (i.e., PHITS) (Sato *et al*. 2018). Figure S12 illustrates the simulation

geometry for exposure to cultured cells (Fig. S12a) and the AB-neutron spectrum used in the PHITS simulation [Fig. S12b]. For calculating the lineal energy spectra (ICRU 1983), the *t-sed* tally of PHITS, which can calculate the distribution of energy deposited in the domain in the macroscopic Monte Carlo simulation, was used (Sato *et al.* 2006, 2009). Figure S13 shows the lineal energy (y in keV/μm) distributions of ^{60}Co γ-rays, AB-neutrons, and BNCT with 20, 200, and 2000 ppm BPA. From the distributions, the y^* value was calculated based on Eq. (2), and the dose–response curve of the cell survival was predicted for the ^{60}Co γ-rays, AB-neutrons, and BNCT in the main paper (Fig. 5d–5f).

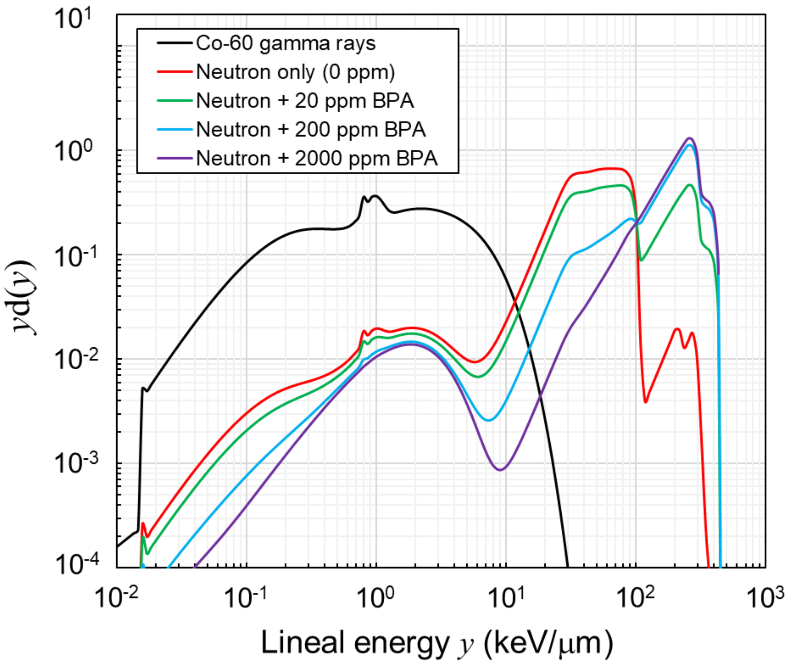

Figure S13. Microdosimetric distribution calculated using the PHITS code: To estimate the RBE values for various radiation exposures, we calculated the lineal energy (y) distributions using the *t-sed* tally, which can calculate the distribution of energy deposited in a domain in the macroscopic Monte Carlo simulation, in the PHITS code. Using the distribution, the MK model enables estimating surviving fractions after various LET irradiations.

The RBE value was also predicted as a function of the administered BPA concentration for the G_1/S and $S/G_2/M$ phases (Fig. S14). The RBE values were calculated using the ratio of the mean inactivation doses (Materials and Methods: Mean Inactivation doses and the RBE). In Fig. S14, the black dashed and colored solid lines represent the model estimation based on the experimental BPA concentration (Fig. 3j) and that assuming that the BPA densities within the HeLa cells are the same as the administered dose (i.e., uptake fraction = 100%), respectively. The ^{60}Co γ-rays were set to be the reference radiation. As shown in S14, the increment of the RBE value by $^{10}B(n,\alpha)^7Li$ reactions in the $S/G_2/M$ phase is higher than that in the G_1/S phase, suggesting the high radiosensitivity for BNCT irradiation, leading to a successful BNCT in the

$S/G_2/M$ phase. Compared with those in the $S/G_2/M$ phase, the HeLa cells in the G_1/S phase exhibit more radioresistance. Based on these, the use of PVA can enhance the RBE value for the G_1/S phase, and cell cycle dependence on boron uptake plays a key role in improving the curative effects of BNCT.

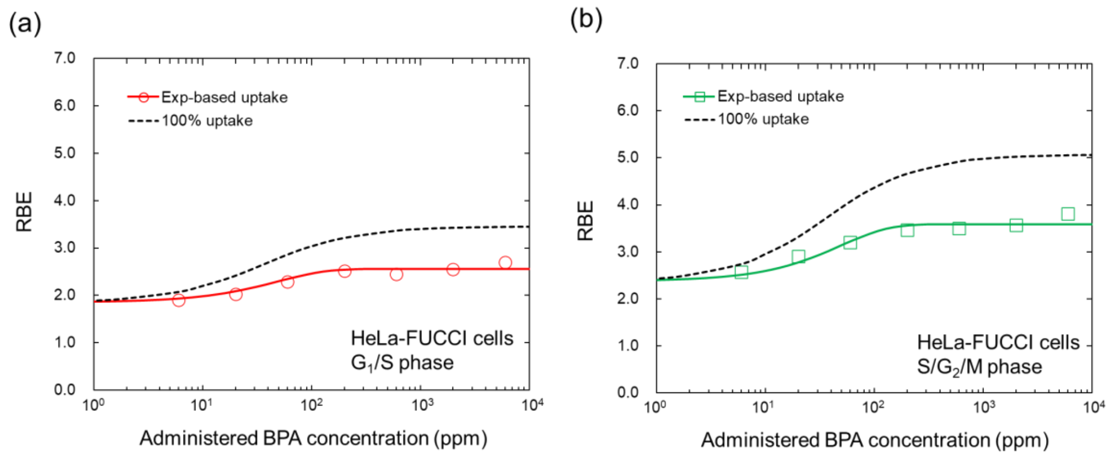

Figure S14. Estimation of the dependence of BPA concentration on RBE value: (a) is that for the HeLa-FUCCI cells in the G_1/S phase, (b) is that for the HeLa-FUCCI cells in the $S/G_2/M$ phase. The RBE_{10} means the relative biological effectiveness (RBE) for 10% cell survival. Note that the ^{60}Co γ-rays were set to be the reference radiation. The colored solid and black dashed lines represent the model estimation based on the experimental BPA concentration (see Fig. 3j) and that assuming that the BPA densities within the HeLa cells are the same as the administered those (i.e., uptake fraction = 100%), respectively.

In the main paper, the dose–response curve of the cell survival in HeLa-FUCCI cells was estimated in the asynchronous phase. The predicted dose–response curve of asynchronous HeLa-FUCCI cells to the corresponding experimental data (Fig. 5c) was compared, and the performance of the present IMK model [Eq. (3)] in the main text) was checked. Thus, in this supplementary file, the RBE value for various cell cycle phases was also predicted using the IMK model [Eq. (3)]. Figure S15 depicts the estimation of the RBE value as a function of the administered BPA concentration for various cell cycle phases. When estimating the RBE, the BPA concentration was used for each cell cycle phase, which was measured in this experiment (Fig. 3). As shown in Fig. S15, the RBE values can be enhanced as the fraction of the G_2/M phase increases. From the estimation results, the cell cycle-specific anticancer agents leading to G_2/M accumulation are expected to enhance the RBE values of BNCT.

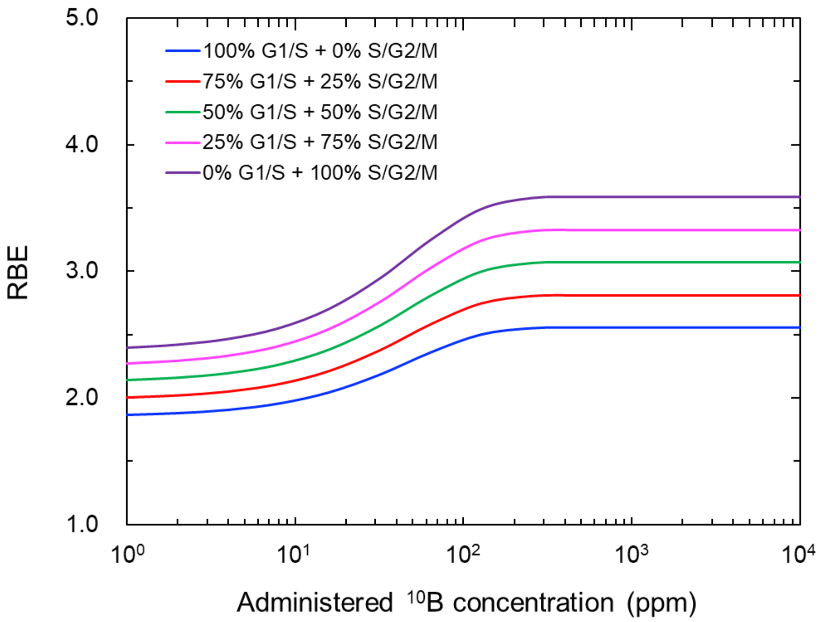

Figure S15. Estimation of the dependence of BPA concentration on RBE value for various cell cycle phases. When calculating the cell survival and the RBE, we used the IMK model (Eq. (3)) based on the experimental BPA concentrations (see Fig. 3j). Note that the ^{60}Co γ-rays were set to be the reference radiation.

As shown in Figs. 5, S14, and S15, the present IMK model allows the estimation of cell killing and the RBE for various cell cycle phases. However, the IMK model [Eq. (3)] for LET dependence was verified in the main text. Herein, a benchmark test was performed by comparing the present model to the experimental RBE values for various ions (i.e., ^{1}H, ^{4}He, ^{6}Li, ^{12}C, ^{16}O, and ^{56}Fe ions).

In the same manner as the calculation methods in the main paper, the y^* value defined in Eq. (2) in the main paper (for calculating z_{1D}^* value) was initially calculated using the PHITS code. Note that $z_{1D}^* = y^*/\rho\pi r_d^2$, where ρ and r_d are the density and radius of the domain, respectively (i.e., ρ = 1.0 g/cm^3 and r_d = 0.5 μm). Second, by employing the z_{1D}^* value and model parameter sets ($\alpha_{0G1/S}$, $\beta_{G1/S}$, $\alpha_{0S/G2/M}$, and $\beta_{S/G2/M}$), the surviving fractions were calculated as a function of absorbed dose using Eqs. (1) and (3). Third, based on Eqs. (4) and (5), the RBE was calculated as a function of LET for ^{1}H, ^{4}He (α-particles), ^{6}Li, ^{12}C, ^{16}O, and ^{56}Fe ions. Figure S16 compares the model predictions and experimental RBE values (Deering & Rice 1962, Goodhead *et al*. 1992, Ito *et al*. 1993, Kato *et al*. 2004, Kaur *et al*. 2013, Li *et al*. 2002) including the Particle Irradiation Data Ensemble (PIDE) database (Friedrich *et al*. 2013). The PIDE is the largest database of cell survival data measured after exposure to ion beams and photon reference radiation. As shown in Fig. S16, the IMK model successfully reproduced the experimental RBE. In the present model, we assumed that the site size r_d = 0.5 μm is a constant value independent of the phase of the cell cycle. Judging from the agreement shown in Fig. S16, the present IMK model is

suitable for estimating the RBE values after high LET ion irradiation (including BNCT irradiations) in various phases of the cell cycle.

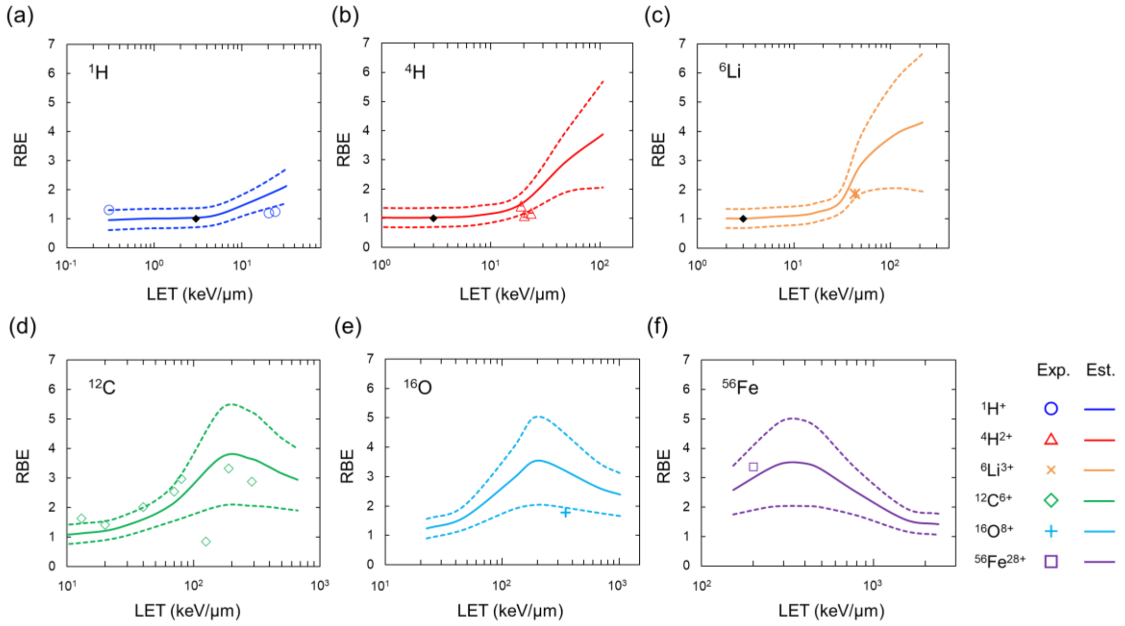

Figure S16. Estimation of the LET-dependence on RBE$_{10}$ value: (a) is the RBE for ^{1}H, (b) is that for ^{4}He (α-particles), (c) is that for ^{6}Li, (d) is that for ^{12}C, (e) is that for ^{16}O, and (f) is that for ^{56}Fe ions. When estimating the RBE value, we used the model parameters listed in Table 1. Note that the ^{60}Co γ-rays were set to be the reference radiation. The experimental RBE data was obtained from a lot of literature (Deering & Rice 1962, Goodhead *et al*. 1992, Ito *et al*. 1993, Kato *et al*. 2004, Kaur *et al*. 2013, Li *et al*. 2002) including the Particle Irradiation Data Ensemble (PIDE) database (Friedrich *et al*. 2013).

As illustrated in Fig. 3F, the experiment shows the low uptake fractions of the HeLa and HeLa-FUCCI cells. To discuss the validity of these experimental results, we tried to compare the experimental radiosensitivity (Davis & Little 1970) to the RBE value estimated based on the IMK model and the measured BPA concentrations (Figs. 3f), as shown in Fig. B6. The experimental data (Davis & Little 1970) was obtained using boron acid, and the boron compound is different from that used in this study (i.e., BPA). Therefore, the comparison is made just for reference. In Fig. S17, the estimated radiosensitivity and RBE values were normalized by that after neutron irradiation without boron administration. From the good agreement between model prediction and the experimental relative radiosensitivity (Fig. S17), the low uptake efficiency of the HeLa and HeLa-FUCCI cells is reasonable.

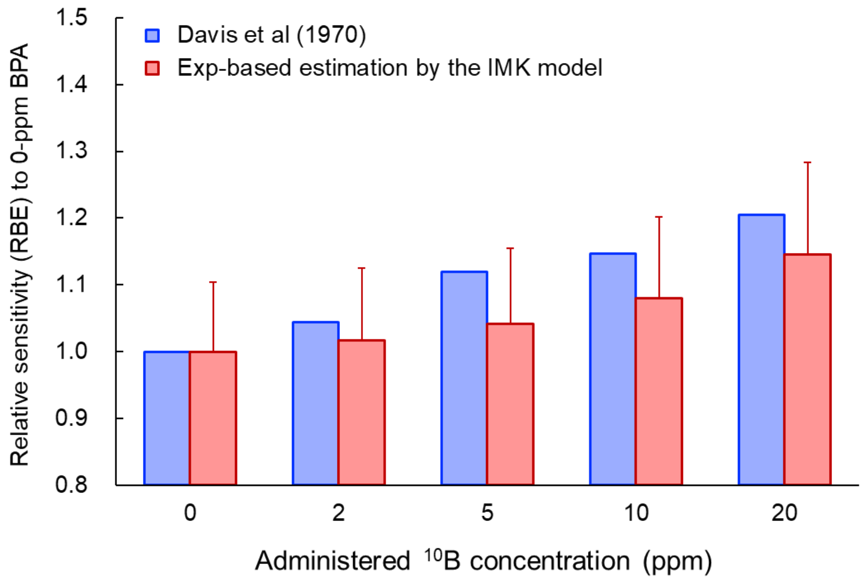

Figure S17. Estimation of RBE and the experimental value using ^{10}B boric acid. In the main manuscript, the experiment showed the saturation of BPA intake and the low intake fraction of the HeLa cells (see Fig. 3f). To discuss the low intake fraction of the HeLa cells, we compared the experimental radiosensitivity (Davis & Little 1970) to the RBE value estimated based on the MK model and the measured BPA density (Fig. 3f). Both of the estimated radiosensitivity and the RBE value by the MK model were normalized by that of 0-ppm BPA administration. As the comparison result, we confirmed that the uptake efficiency of the HeLa cells is low.

References

Davis MA, Little JB 1970 Relative Biological Effectiveness of the ^{10}B(n,α)^{7}Li Reaction in HeLa Cells. *Radiat. Res.* 43, 534–553.

Deering RA, Rice R 1962 Heavy Ion Irradiation of HeLa cells. Radiat. Res. 17, 774–786.

Fantidis JG 2018 Beam shaping assembly study for BNCT facility based on a 2.5 MeV proton accelerator on Li target. *J. Theo. Appl. Phys.* 12, 249–256.

Friedrich T, Scholz U, Elsässer T, Durante M, Scholz M 2013 Systematic analysis of RBE and related quantities using a database of cell survival experiments with ion beam irradiation. *J. Radiat. Res.* 54(3), 494–514.

Goodhead DT, Belli M, Mill AJ et al 1992 Direct comparison between protons and alpha-particles of the same LET. I: irradiation methods and inactivation of asynchronous V79, HeLa and C3HT1/2 cells. *Int. J. Radiat. Biol.* 61, 611–624.

Hawkins RB 1996 A microdosimetric-kinetic model of cell death from exposure to ionizing radiation of any LET, with experimental and clinical applications. *Int. J. Radiat. Boil.* 69, 739–755.

ICRU 1983 Microdosimetry; Report 36; International Commission on Radiation Units and Measurements: Rockville, MD, USA.

Inaniwa T, Suzuki M, Furukawa T, Kase Y, Kanematsu N, Shirai T, Hawkins RB 2013 Effects of Dose-Delivery Time Structure on Biological Effectiveness for Therapeutic Carbon-Ion Beams Evaluated with Microdosimetric Kinetic Model. *Radiat. Res.* 180, 44–59.

Ito H, Yamashita S, Nishiguchi I, Ka W-J, Hashimoto S, Yatagai F, Kanai T 1993 Carbon Beam Irradiation of Monolayer Cells. *Nippon Acta. Radiol.* 53, 321–328.

Kato AT, Tsuda A, Uesaka M, Fujimori A, Kamada T, Tsuji H, Okayasu R 2011 *In vitro* characterization of cells derived from chordoma cell line U-CH1 following treatment with X-rays, heavy ions and chemotherapeutic drugs. *Radiat. Oncol.* 6, 116.

Kase Y, Yamashita W, Matsufuji N, Takada K, Sakae T, Furusawa Y, Yamashita H, Murayama S 2013 Microdosimetric calculation of relative biological effectiveness for design of therapeutic proton beams. *J. Radiat. Res.* 54, 485–493.

Kaur H, Pujari G, Semwal MK, Sarma A, Avasthi DK 2013 In vitro studies on radiosensitization effect of glucose capped gold nanoparticles in photon and ion irradiation of HeLa cells. *Nucl. Instr. Methods Phys. Res. Sect. B* 301, 7–11.

Li W-J, Zhou G-M, Wei Z-Q, Wang J-F, Dang B-R, Li Q, Xie H-M 2002 RBE of Cells Irradiated by Carbon Ions. High Ener. Phys. Nucl. Phys. 26(7), 742–746.

Matsuya Y, Kimura T, Date H 2017 Markov chain Monte Carlo analysis for the selection of a cell-killing model under high-dose rate irradiation. *Med. Phys.* 44, 5522–5532.

Matsuya Y, Fukunaga H, Omura M, Date H 2020 A Model for Estimating Dose-Rate Effects on Cell-Killing of Human Melanoma after Boron Neutron Capture Therapy. *Cells* 9(5), 1117.

Saga R, Matsuya Y, Sato H, Hasegawa K, Obara H, Komai F, Yoshino H, Date H, Aoki M, Hosokawa Y 2023 Translational study for the clinical outcome of stereotactic body radiotherapy on non-small cell lung cancer based on cell-killing model considering cancer stem-like cells. *Radiother. Oncol.* 181, 109444.

Sato T, Watanabe R and Niita K 2006 Development of a calculation method for estimating specific energy distribution in complex radiation fields. *Radiat. Prot. Dosim.* 122, 41–45.

Sato T, Kase Y, Watanabe R, Niita K, Sihver L 2009 Biological Dose Estimation for Charged-Particle Therapy Using an Improved PHITS Code Coupled with a Microdosimetric Kinetic Model. *Radiat. Res.* 171, 107–117.

Sato T, Iwamoto Y, Hashimoto S, Ogawa T, Furuta T, Abe S, Kai T, Matsuya Y, Matsuda N, Hirata Y, Sekikawa T, Yao L, Tsai P-E, Ratliff HN, Iwase H, Sasaki Y, Sugihara K, Shigyo N, Sihver L, Niita K. Recent improvements of the particle and Heavy Ion transport code system – PHITS version 3.33. *J. Nucl. Sci. Technol.* (2018), doi: 10.1080/00223131.2023.2275736.

Seino R, Uno H, Prise KM, Fukunaga H 2023 Cell Cycle Dependence of Cell Survival following Exposure to X-rays in synchronous HeLa Cells expressing Fluorescent Ubiquitination-based Cell Cycle Indicators. Under review

Takada K, Sato T, Kumada H, Koketsu J, Takei H, Sakurai H, Sakae T 2018 Validation of the physical and RBE-weighted dose estimator based on PHITS coupled with a microdosimetric kinetic model for proton therapy. J. Radiat. Res. 59(1) 91–99.
